# From flux analysis to self contained cellular models

**DOI:** 10.3389/fsysb.2025.1546072

**Published:** 2025-08-22

**Authors:** Andreas Kremling

**Affiliations:** Systems Biotechnology, School of Engineering and Design, Technical University of Munich, Munich, Germany

**Keywords:** flux balance analysis, coarse-grained models, thermodynamics, resource allocation, enzyme kinetics

## Abstract

Mathematical models for cellular systems have become more and more important for understanding the complex interplay between metabolism, signalling, and gene expression.In this manuscript, starting from the well-known flux balance analysis, tools and methods are summarised and illustrated by various examples that describe the way to models with kinetics for individual reactions steps that are finally self-contained. While flux analysis requires known (measured) input fluxes, self-contained (or self-sustained) models only get information on concentrations of environmental components. Kinetic reaction laws, feedback structures, and protein allocation then determine the temporal output of all intracellular metabolites and macromolecules. Emphasis is placed on (i) mass conservation, a crucial system property frequently overlooked in models incorporating cellular structures like macromolecular structures like proteins, RNA, and DNA, and (ii) thermodynamic constraints which further limit the solution space. Matlab Live Scripts are provided for all simulation studies shown and additional reading material is given in the appendix.

## 1 Introduction

Understanding complex systems, both in technical and non-technical sciences, relies heavily on mathematical modeling. This holds true for life sciences, particularly in systems biology, where mathematical modeling serves as a formalization tool for comprehending the intricacies of a system. Systems theory provides a framework for constructing models with various dimensions. One dimension pertains to the level of detail in the model, spanning from simple qualitative interaction networks to extensive, mass-conservative quantitative models detailing processes within cells and their fluctuating environments. Another dimension involves whether the model represents an average cell or individual cells within an environment. Modeling individual cells demands sophisticated approaches, such as employing population balance equations developed by Ramkrishna and colleagues (Ramkrishna, 2000) or adopting an ensemble modeling approach. Both methods necessitate distinct numerical schemes for resolution. Additional dimensions encompass whether the system is static or dynamic, and if the model requires structural elements, like an objective function, to explore the potentially infinite solution space.

The text at hand endeavors to initiate the analysis of a biochemical reaction network using flux balance analysis (FBA), a well-established method in cellular systems modeling. Therefore, only a basic understanding of reaction engineering is needed, while in-depth knowledge of microbial physiology commonly applied in biotechnology is unnecessary, since the examples are based on a simplified (toy) network. It concludes by constructing self-contained models that consider cellular macromolecular units linked to metabolism. Emphasis is placed on mass conservation, a crucial system property frequently overlooked in models incorporating cellular structures, leading to an incorrect degree of freedom for selecting system unknowns. While standard FBA typically considers a single biomass reaction, the proposed framework accommodates the structured nature of cells, highlighting resource allocation importance. Simple, self-contained models derived from this framework serve as a foundation for more intricate models.

A systematic procedure is presented, starting with a basic biochemical network devoid of macromolecular units. It delves into thermodynamic properties and introduces methods for a proper description avoiding inconsistencies with physical basics. Conforming to the dimensions mentioned earlier, the manuscript confines itself to detailed quantitative models for an average cell, analyzing static properties.

Therefore, it is not only suitable for beginners with basic background in mathematics (algebraic knowledge and differential calculus is required) and modeling but also for advanced students who could deepen their knowledge, especially when the macromolecular unit structure of the cell is introduced (here two different approaches are used).

After studying the text, the reader gains an understanding of the fundamental mathematical framework underlying models of cellular systems, as introduced in Section 2.1. Although a simplified (toy) model is used for illustration, comprehensive stoichiometric models of real cellular systems are available in specialized databases and can serve as a foundation for further exploration. Section 2.2 addresses basic thermodynamic challenges, enabling the reader to formulate systems of equations that avoid thermodynamic inconsistencies. Section 2.3 expands the discussion to include cellular models that incorporate macromolecular structures. A key outcome here is the development of objective functions that account not only for intracellular fluxes but also for the enzyme requirements necessary to sustain those fluxes. Section 2.4 introduces an extended equation system that integrates a larger set of macromolecular components and explores their potential roles in feedback mechanisms—such as how proteome allocation can influence intracellular flux distributions. Finally, the text introduces the reader to kinetic modeling approaches (Section 2.5). Whereas fluxes were previously treated as unknown variables, this section presents kinetic equations for individual enzymatic reactions. This allows for the analysis of the dynamic relationship between metabolic fluxes and intracellular metabolite concentrations, illustrated through the ongoing example of a small metabolic network.

Last but not least, hopefully lecturers and also well-advanced scientist will find interesting examples for their courses. A quantitative description always aims for a better understanding of the system at hand. Although the examples used are small, the presented methodology is general and can be applied to larger stoichiometric networks to determine intracellular flux distributions given measured uptake and production rates. Moreover, problems of resource allocation can be considered and will allow researchers to compare simulation results with own data. Larger differences should lead to the formulation and testing of new hypotheses that again will lead to new sets of experimental experiments. Additional reading material (books and standard publications) are provided in the Supplementary Appendix.

The examples provided adhere strictly to quantitative principles, employing physiologically meaningful parameters and standard units such as g grams, mol moles, gDW grams of dry weight (gDW) , and h hours for time constants. All simulations are executed using Matlab; live-scripts are provided for replicating the examples. Although software packages are available that support the reader by implementing models and performing simulations studies, only standard Matlab code is provided here. The advantage from the learning perspective is that the scripts can be directly used in the lecture or exercises. In typical courses, the focus is on the modeling approaches and how they are used to solve problems in systems biology and metabolic engineering. More details on available software tools can be found in the Supplementary Appendix.

## 2 Case studies

### 2.1 Mass balance in biochemical reaction systems

A common feature of all deterministic models that were developed for applications in systems biology, medical systems biology, or metabolic engineering is mass conservation. This is valid on the level of single bio-chemical reactions but also for larger networks to whole-cell models. For the studies presented here, mass conservation is obtained for single biochemical reactions which also result in mass conservation on the level of biomass synthesis; closely related to this, it allows a clear calculation of the specific growth rate μ if cell division is considered. The specific growth rate is defined as the change of biomass $m_{X}$ over time divided by the biomass $\mu =\frac{dm_{X}/dt}{m_{X}}$ (often in literature, a definition based on the concentration of biomass $c_{X}$ , that is, the ratio of biomass to the volume in the bioreactor system $c_{X}=m_{X}/V$ , is found that will, however, lead to inconsistencies when considering processes with a changing volume). With biomass, the weight of all cells in the experimental set-up is meant; the models developed in the text are based on a defined structure with metabolites and macromolecular units. To be mass conservative, the mass fraction of all metabolites and units (given in g/g ) must add up to 1g .

A standard notation with stoichiometric coefficients $\gamma_{i}$ and reaction rate r for a single reaction reads:

$$\left|\gamma_{A}\right|A+\left|\gamma_{B}\right|B\;\overset{r}{⇌}\;\left|\gamma_{C}\right|C+\left|\gamma_{D}\right|D$$ (1)

The numbers given in front of the metabolites are positive numbers (therefore they are written with value sign |•| ) and describe the number of molecules involved in the reaction on a molar basis. They report on the left side the number of molecules that serves as substrates, and on the right side the number of molecules that serves as products. In the current form, these terms cannot be used for further calculations and must be translated into equations. As a general agreement, the stoichiometric coefficients on the left side are taken as negative values ( $\gamma_{A}$ and $\gamma_{B}$ ), while the coefficients on the right side are positive ( $\gamma_{C}$ and $\gamma_{D}$ ). The corresponding equation reads as follows:

$$\underset{{\underline{n_{i}}}^{T}}{\underbrace{\left(\begin{array}{cccc} \gamma_{A} & \gamma_{B} & \gamma_{C} & \gamma_{D} \end{array}\right)}}\left(\begin{array}{c} A\\ B\\ C\\ D \end{array}\right)=0$$ (2)

with the vector $\underline{n_{i}}$ is the stoichiometric vector of the reaction i . However, since the equation is only based on the numbers of the interacting molecules, we cannot infer, if the mass balance is closed. Here, the molecular weight $w_{j}$ of compounds j come into play. A closed balance appears if we multiply the stoichiometric vector with the vector of the molecular weights $\underline{w}$ of the metabolites:

$${\underline{w}}^{T}\underline{n_{i}}=0$$ (3)

and as a results, a value of zero appears on the right side (scalar value since a row vector is multiplied with a column vector).

Since in a cell, a large number of reactions run simultaneously, the stoichiometric coefficients are stored in a matrix N , called stoichiometric matrix (each reaction is represented in a column):

$$N=\left(\begin{array}{ccc} {\underline{n}}_{1} & {\underline{n}}_{2} & \cdots \end{array}\right)$$ (4)

For l compounds and q reactions, the dimension of N is l×q ^1^. Example 1

In this example, the notation for a chemical reaction is applied for a single reaction. A reaction equation reads ( $\gamma_{A}=-2$ , $\gamma_{B}=1$ , $\gamma_{C}=2$ ):

$$2A\;\overset{r}{⇌}\;1B+2C$$ (5)

and with the molecular weights $w_{A}=30g/mol$ , $w_{B}=20g/mol$ , and $w_{C}=20g/mol$ , we see that Equation 3 is exactly fulfilled.

Variable r is used to describe the velocity of the reaction. Important parameters that are introduced later on to describe this term are enzyme specific parameters like the turnover number $k_{\mathrm{cat}}$ , substrate and product binding constants $K_{S}$ , $K_{P}$ , and the equilibrium constant $K_{Eq}$ . Furthermore, the reaction rate also depends on the concentration of the reaction partners. In this way, r could depend on a long list of concentrations and kinetic paramters $r\left(c_{1},c_{2},\cdots k_{1},k_{2},\cdots K_{1},K_{2}\cdots \;\right)$ .

Mass conservation on the level of single reactions implies that also for each compound in the system, an equation can be written down that sums up all processes that either increase or decrease the mass (or number of molecules) of the compound. Unfortunately, we cannot write down directly an equation $m_{j}\left(t\right)$ that describes the course of the mass of the compound j over time t ; rather we can sum up all mass flow over time that changes the mass either in a positive or a negative way. The change of a compound over time is expressed with dm/dt and the notation used in the engineering community $\dot{m}$ is used in the following. This results in a ordinary differential equation (o.d.e.) for the mass $\dot{m_{j}}$ of the compound or $\dot{n_{j}}$ if we consider the molar number of compound j . Typically, only biochemical conversion due to reactions is considered that includes uptake from the environment, excretion into the environment, or conversion into another compounds in the environment or inside the cell. Although diffusion is important in larger cells, it is not considered here.

The change of the mol number n – that is proportional to its mass m – of a compound is given by:

$$\dot{n_{j}}=\underset{i}{\sum }\;\gamma_{ji}\;r_{i}\;m_{X};$$ (6)

That is, the reaction velocity $r_{i}$ is weighted with stoichiometric coefficients $\gamma_{ji}$ and is multiplied with the total biomass $m_{X}$ . Note, that the sum is taken of all reactions with index i for component with index j (therefore, the stoichiometric coefficients always have two indexes). This is necessary, since, typically, a reaction velocity $r_{i}$ is given as a specific rate, that is, based on the total biomass with unit [mol/gDWh] . In principle, we are done, and we could continue with the analysis of systems with a given number of reactions and compounds. However, in the current form on the left hand side, the change of numbers per time unit is given while on the right side reaction velocities appears that might depend on the concentration c of the compounds $r_{i}=r_{i}\left(c_{1},c_{2},\cdots \;\right)$ . Therefore, a further step is needed here: To convert the left hand side, intracellular concentrations are defined by $c_{j}=n_{j}/m_{X}$ with unit [mol/gDW] , that is, based on the total biomass. Starting from $n=c\cdot m_{X}$ and using the product rule for the derivative for $\dot{n}=\dot{c}m_{X}+\dot{m_{X}}c$ , the final equation reads:

$${\dot{c}}_{j}=\underset{i}{\sum }\gamma_{ji}r_{i}-\frac{c\dot{m_{X}}}{m_{X}}=\underset{i}{\sum }\gamma_{ji}r_{i}-\mu c_{j}$$ (7)

with the specific growth rate μ that appears as dilution term which can be understood as follows: Assume that all rates that synthesize or degrade the compound are zero, the concentration of the compound will not stay constant but will go to zero due to cell growth (in every cell devision, the biomass is doubled). Equation 7 can be written for all compounds in the cell taking into account all reactions stored in the stoichiometric matrix N . In this way, all stoichiometric coefficients for a single metabolite appear in the respective row of matrix N .

$$\underline{ċ}=N\underline{r}\left(\underline{c}\right)-\mu \underline{c}$$ (8)

Based on this fundamental equation, model analysis can be started. A standard tool here is the determination of all fluxes in a given metabolic network. Flux analysis focuses on balanced states, that is, it is assumed that all processes result in a quasi steady-state of the involved compounds. Furthermore, taking into account that in the upper equation the last summand has numeric values of different orders of magnitude, the standard equation for flux analysis simplifies:

$$\underline{0}=N\underline{r}-\mu \underline{c}\;\overset{r_{i}\gg \mu c}{\Rightarrow }\;\underline{0}=N\underline{r}$$ (9)

Please note, that the dependencies of $r_{i}$ on the concentration vector $\underline{c}$ is omitted here. In this way, the reaction rates $r_{i}$ are the unknowns of the equation system. Since the number of reactions rates (the column of the matrix N ) is usually higher than the number of metabolites (the rows of the matrix N ), the set of possible solutions of Equation 9 is infinite, and, usually is expressed in form of the null space K of N (null space vectors are orthogonal to each row, and therefore fulfill Equation 9 (see Supplementary Appendix). Since all linear combinations of null space vectors also represent solutions, some solutions are characterized by the following property: it is not possible to omit one of the reactions with a nonzero flux while for the remaining reactions, a different solution of Equation 9 can be found, or in other words, the solution cannot be decomposed. These special solutions are called elementary flux modes.

Example 2

For the bacterium *Escherichia coli*, the uptake rate for glucose in a minimal medium is estimated to be 6mmol/gDWh for a growth rate of μ=0,51/h . High values for intracellular concentrations for metabolites in central pathways are reported to be 10mM=0,03mmol/gDW ^2^, and μ⋅c≈0,015mmol/gDWh . The dilution term is a factor 400 smaller than the uptake rate and it is justified to neglect it in this case.

Equation 9 will be the starting point for the analysis of our first network. One can assume that not all of the rates $r_{i}$ are unknown; typically good measurable rates are the carbon source, oxygen, ${\text{CO}}_{2}$ , and nitrogen uptake. This allows us to split the rate vector into unknown rates (index u ) and known rates (index k ):

$$\underline{0}=N\underline{r}=N_{u}{\underline{r}}_{u}+N_{k}{\underline{r}}_{k}$$ (10)

and the number of unknowns is reduced. In general the number of rows corresponds to the number l of compounds and the dimension of matrix N is l×q with q reactions.

Formally, we are looking for a vector $\underline{r}$ or ${\underline{r}}_{u}$ that solves the Equation 10; this solution is called a flux distribution or a flux map. The number of solutions strongly depends on structural properties of matrix N : in the cases considered here (for cellular networks, l≪q is valid), there are a infinite number of solutions^3^. Therefore, to find a physiological meaningful solution, one solution is selected that fulfils additional properties. One possibility is to choose a solution that maximizes or minimizes an objective function. Objective functions are chosen in such a way that physiological criteria are met, for example, bacterial systems tend to generate ATP as much as possible, grow as fast as possible or behave in a economic positive way in conditions with substrate surplus or scarcity (Schuetz et al., 2007)^4^. Mathematically, in this way, an optimization program is defined:

$$\begin{array}{ccc} \max f\left(\text{or}\min f\right): & {\underline{c}}^{T}\underline{r}= & g_{1}r_{1}+g_{2}r_{2}+\cdots g_{n}r_{n}\\ s.t. & & \\ N_{u}{\underline{r}}_{u}= & -N_{k}{\underline{r}}_{k}\\ & r_{i,min}\leq r_{u,i} & \leq r_{i,max} \end{array}$$ (11)

with entries $g_{i}$ that select a single rate or combinations of rates to be extremal, and two “subject to” equations: constraints from the mass balance and boundaries for the fluxes.

### 2.2 Flux models without drain into biomass

**FIGURE 1:**
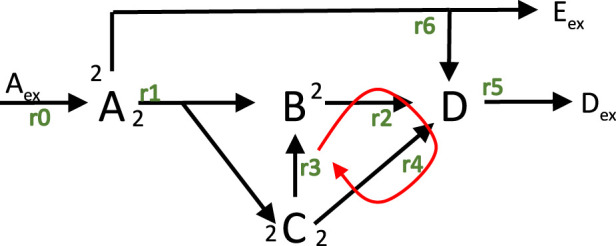
Network structure with input rate $r_{0}$ and six unknown fluxes; reactions $r_{1,2,3,4}$ are internal one; reactions $r_{2,3,4}$ build a cycle. Reactions $r_{5,6}$ describe the exchange with the environment.

On the first level, a simple network is considered, and the basic procedure to determine the flux maps is introduced. On a later stage, biomass synthesis will be considered. For the following examples, the stoichiometric matrix N reads (the external components are not considered, therefore, there are only four rows and seven columns):

$$N=\left(\begin{array}{ccccccc} 1 & -2 & 0 & 0 & 0 & 0 & -2\\ 0 & 1 & -2 & 1 & 0 & 0 & 0\\ 0 & 2 & 0 & -1 & -2 & 0 & 0\\ 0 & 0 & 1 & 0 & 1 & -1 & 1 \end{array}\right)$$ (12)

and is illustrated in Figure 1.

Rates are numbered from $r_{0}$ (input flux) to $r_{i},\;i=1,6$ . In this network, the number of reactions exceeds the number of components, and one can see that the rank (see Supplementary Appendix) of N is r=4 and that the dimension of the null space (or kernel) of the matrix is k=3 . For the example, already a number of options are given, to select one flux distribution as optimal. In all cases, the input flux is fixed $\left(r_{0}=1mol/gDWh\right)$ .

The calculation of flux distributions is shown for a number of different settings in Example 3. First, two different objective functions are applied and the different flux maps are compared Example 3a,b. For a selected objective function in Example 3c,it turns out, that some fluxes reach its maximal value. A closer inspection of the flux map reveals that thermodynamic laws are violated. After introducing the basic equations for a thermodynamic analysis for a single reaction and for biochemical reaction network, Example 3c is considered again and is now solved taking into account the presented equations.

Example 3

**FIGURE 2:**
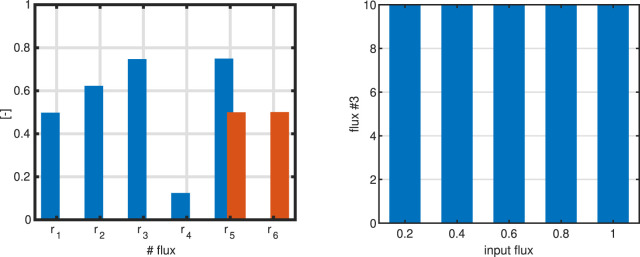
Left: Bar plot to compare a single flux objective $\left(r_{5}\right)$ with sum of fluxes objective function (case 3a/b). As input flux, one unit is used. Right: Value of the objective function if $r_{3}$ is the objective function (case 3c) for different values of the input flux (five different input fluxes are used).

3a. Rate $r_{5}$ is selected to be maximized. 3b. Sum of all fluxes is minimized. 3c. Rate $r_{3}$ is maximized. Optimal solutions for seven different settings are shown in Figure 2. The flux maps differ significantly; in case 3a, all of the incoming flux into compound A could be converted into $D_{ex}$ . In case 3b, we note, that minimizing the sum of all fluxes, results in only two fluxes that were active $\left(r_{5,6}\right)$ while all other fluxes are zero. From all possible elementary flux modes for this system (shown in the Supplementary Appendix), the calculated flux distribution has overall the minimal sum.

For 3c we note that–independent from the input flux–the objective always shows the same value. This value corresponds to the upper limit that was set by the boundaries. Boundaries limit the solution space and are based on (experts) knowledge on the system; they could be positive or negative, depending on the problem formulation. Since the direction of the fluxes are not known before in general, positive and negative values are used. How can we interpret this result? A closer and detailed look in this case reveals that not only flux $r_{3}$ shows a special behaviour but also fluxes $r_{2}$ and $r_{4}$ . Both change their values according to match Equation 10. This means that the fluxes are running in a cycle, represented by the three rates shown in Figure 1.

#### 2.2.1 Thermodynamics

To solve the problem mentioned in the last example, thermodynamic considerations come into play. For reaction systems, the change of the free Gibbs energy ΔG is of most importance here. The free Gibbs energy can be formulated in two ways; in the first case, it depends on the chemical potential $\mu_{j}$ of the compounds, in the second case, the concentrations of the compounds $c_{j}$ are directly used and replace the chemical potential (for simplicity, letter c is omitted and a capital letter for the concentration of the compound is used instead). For a single reaction we get with a value $\Delta G_{i}^{0^{\prime }}$ under standard conditions (pH = 7, T = 298 K):

$$\Delta G_{i}=\Delta G_{i}^{0^{\prime }}+RT\ln\frac{\prod P^{\gamma_{p}}}{\prod S^{\gamma_{s}}}$$ (13)

with index p is used for the products of the reaction (written on the right side of the reaction equation), index s is used for the substrates (written on the left side of the reaction equation), and R is the gas constant. Numerical values for ΔG are as follows:

• ΔG<0 in case the reaction is running in given direction from substrates to products, that is, reaction rate $r_{i}>0$ .
• ΔG=0 for reactions that are in equilibrium, that is, the net reaction rate (difference between forward and backward reaction) is zero. For this case, a relationship between $\Delta G_{i}^{0^{\prime }}$ and the equilibrium constant $K_{eq}$ of the reaction can be obtained:

$$0=\Delta G_{i}^{0^{\prime }}+RT\ln\frac{\prod \overset{\gamma_{p}}{\underset{Eq}{P}}}{\prod \overset{\gamma_{s}}{\underset{Eq}{S}}}\Rightarrow \Delta G_{i}^{0^{\prime }}=RT\ln\frac{1}{K_{eq}}.$$ (14)

This important equation relates the equilibrium constant of a reaction directly to the standard value of the Gibbs free energy.

• ΔG>0 in case the reaction is running in the reversed direction from products to substrates, that is, reaction rate $r_{i}<0$ .

Example 4

For a reaction with one substrate and two products (*cf.* Example 1):

$$2A\;\overset{r}{⇌}\;1B+2C$$ (15)

The forward and the backward reaction are given by $r_{f}=k^{+}A^{2}$ , $r_{b}=k^{-}BC^{2}$ , if simple kinetic rate laws (mass action) are applied. Here the reaction rate is proportional to the involved metabolites. Setting forward rate and backward rate equal, one get:

$$k^{+}A^{2}=k^{-}BC^{2}\Rightarrow K_{eq}=\frac{k^{+}}{k^{-}}=\frac{B_{Eq}{C_{Eq}}^{2}}{{A_{Eq}}^{2}}$$ (16)

In this case, Equation 13 reads:

$$\Delta G=0=\Delta G^{0^{\prime }}+RT\ln\frac{B_{Eq}{C_{Eq}}^{2}}{{A_{Eq}}^{2}}$$ (17)

and one obtains finally by combining the last two equations:

$$0=\Delta G^{0^{\prime }}+RT\ln K_{Eq}\Rightarrow \Delta G^{0^{\prime }}=RT\ln\frac{1}{K_{eq}}$$ (18)

If $K_{Eq}>1$ , that is, $k^{+}>k^{-}$ , the standard value is negative (the log is smaller than zero for values smaller than 1), indicating that under standard conditions, the reaction would take place from left to right. Reaction kinetics could be more sophisticated in case that enzymes act as catalysts (see below).

Taking advantage of the logarithmic function, the exponents can be written before the ln symbol; furthermore, using matrix operations, Equation 13 can be re-written in a scaled nomenclature for all internal reactions of the network using the stoichiometric matrix $N^{\prime }$ and vector $\underline{x}$ representing the logarithmic values of the concentrations $x_{j}=\ln c_{j}$ :

$${\underline{\Delta G}}^{*}=\frac{\underline{\Delta G}}{RT}=\frac{{\underline{\Delta G}}^{0^{\prime }}}{RT}+N^{\prime T}\underline{x}$$ (19)

with ${\underline{\Delta G}}^{*}$ is the vector with entries of the scaled free Gibbs energies of the reactions (that is divided by the term RT ). Only internal reactions are taken into account, since information on external concentrations of metabolites are not considered here.

From what we have seen before, a simple relationship between fluxs $r_{i}$ and $\Delta G_{i}$ values is obtained:

$$\Delta G_{i}^{*}r_{i}<0,$$ (20)

That is, the product of the free Gibbs energy and the respective flux is always negative. For more than one reaction, the following notation is used:

$${\underline{\Delta G}}^{*}•\underline{r}<\underline{0}.$$ (21)

#### 2.2.2 Thermodynamics in networks

At this point, we have to note, that the values of ${\underline{\Delta G}}^{0^{\prime }}$ and therefore also the equilibrium constants are not independent in a network. If we consider again the cycle with reactions $r_{2}$ , $r_{3}$ , $r_{4}$ we see two ways from C to D ; the first one directly via $r_{4}$ and the second via $r_{3}$ and $r_{2}$ (Figure 1). In other words, one can think on water that falls down a fall on two ways, however, at the bottom the energy status for both ways is equal and it is not possible to bring back the water on top without bringing in energy.

In the last section, we have seen that fluxes are coupled with metabolic concentrations via the Gibbs free energy. Considering large networks, this would require additional state variables (either the Gibbs free energy $\Delta G_{i}$ for reaction i , the chemical potential $\mu_{j}$ for component j , or the concentration $c_{j}$ itself) to obtain a valid flux distribution. In the following different ideas are introduced to guarantee that the fluxes are not running in a cycle. The meaning of cycle is strictly limited here to thermodynamic considerations; other types of cycles such as futile cycles or control loops which are thermodynamically feasible are not meant.

As a starting point, an equilibrium is considered, that is, the net rate of all fluxes is zero as well as the three ΔG values. From above, the following set of equations is obtained (the equations are valid only in equilibrium, here we omit index Eq ):

$$\begin{array}{ccc} \Delta G_{2}^{*}=0\Rightarrow \Delta G_{2}^{*0^{\prime }}+\ln\frac{c_{D}}{c_{B}^{2}}\Rightarrow \frac{c_{D}}{c_{B}^{2}}=e^{-\Delta G_{2}^{*0^{\prime }}} & & \\ \Delta G_{3}^{*}=0\Rightarrow \Delta G_{3}^{*0^{\prime }}+\ln\frac{c_{B}}{c_{C}}\Rightarrow \frac{c_{B}}{c_{C}}=e^{-\Delta G_{3}^{*0^{\prime }}} & & \\ \Delta G_{4}^{*}=0\Rightarrow \Delta G_{4}^{*0^{\prime }}+\ln\frac{c_{D}}{c_{C}^{2}}\Rightarrow \frac{c_{D}}{c_{C}^{2}}=e^{-\Delta G_{4}^{*0^{\prime }}} & & \end{array}$$ (22)

and one can see by close inspection:

$$-\Delta G_{2}^{*0^{\prime }}-2\Delta G_{3}^{*0^{\prime }}=-\Delta G_{4}^{*0^{\prime }}\Rightarrow -\Delta G_{2}^{*0^{\prime }}-2\Delta G_{3}^{*0^{\prime }}+\Delta G_{4}^{*0^{\prime }}=0$$ (23)

The equation can be re-written with the equilibrium constants of the reactions (Equation 18):

$$K_{\mathrm{Eq2}}K_{\mathrm{Eq3}}^{2}=K_{\mathrm{Eq4}}$$ (24)

Can we obtain these equations also in a more systematic way? The cycle given by the three reactions can be obtained by the following equation; if only internal reactions are considered, that is, there is no exchange of mass with the environment, then, the null space K of matrix $N^{\prime }$ represents the reactions of the cycles in the network. In our case, the matrix of internal reaction reads:

$$N^{\prime }=\left(\begin{array}{cccc} -2 & 0 & 0 & 0\\ 1 & -2 & 1 & 0\\ 2 & 0 & -1 & -2\\ 0 & 1 & 0 & 1 \end{array}\right)\Rightarrow N^{\prime }K=\underline{0}$$ (25)

and one obtains for the null space:

$$K=\left(\begin{array}{c} 0\\ -1\\ -2\\ 1 \end{array}\right)$$ (26)

Finally, we see that

$$K^{T}{\underline{\Delta G}}^{*0^{\prime }}=0$$ (27)

is exactly Equation 23.

The last equation is not only true for the equilibrium. The Gibbs free energy can be written as a function of the stoichiometric matrix $N^{\prime }$ and the vector of the chemical potential $\underline{\mu }$ :

$${\underline{\Delta G}}^{T}={\underline{\mu }}^{T}N^{\prime }$$ (28)

Starting from the observation from above that the null space vector K of $N^{\prime }$ contains all cycles in the network, the following relationship is valid:

$$N^{\prime }\underline{r}=0$$ (29)

A multiplication of the last equation from the left side with the vector $\underline{\mu }$ gives:

$${\underline{\mu }}^{T}N^{\prime }\underline{r}=0\Rightarrow {\underline{\Delta G}}^{T}\underline{r^{\prime }}=0\Rightarrow {\underline{\Delta G}}^{T}K=\underline{0}$$ (30)

That is, the values for $\underline{\Delta G}$ must fulfill additional constraints; a multiplication with all vectors in the null space K of $N^{\prime }$ must be zero.

Example 5

Considering the loop in the network above, one gets:

$$K^{T}\underline{\Delta G}=-\Delta G_{2}-2\Delta G_{3}+\Delta G_{4}=0$$ (31)

Now, assume that in the solution flux $r_{4}$ is zero, that is $\Delta G_{4}=0$ ; as a result, we get the constraint from the last equation:

$$\Delta G_{2}=-2\Delta G_{3}$$ (32)

In this case, a solution with a flux from C to B via reaction $r_{3}$ and $r_{2}$ with positive values is forbidden; if there is no flux between C and D due to a reaction equilibrium, this is also true for every different way from C to D .

To incorporate these conditions in the linear framework used so far, we take advantage of a method described by Schellenberger et al. (2011). The methods uses integer variables $z_{i}$ for each reaction/Gibbs energy with values only $z_{i}=0$ or $z_{i}=1$ . One difficulty given by the equations above is, that a vector with zero entries for the $\Delta G_{i}$ values is always a solution to Equation 30 which is, however, undesired. To overcome this, the following equation system excludes value equal zero for the ΔG vector:

$$\begin{array}{ccc} -M_{i}\left(1-z_{i}\right)\leq & r_{j} & \leq M_{i}z_{i}\\ -M_{i}z_{i}+\epsilon_{i}\left(1-z_{j}\right)\leq & \Delta G_{i} & \leq -\epsilon_{i}z_{i}+M_{i}\left(1-z_{i}\right) \end{array}$$ (33)

with $\epsilon_{i}$ is a small number while $M_{i}$ is a sufficiently large number. For the two cases (i) $z_{i}=0$ we obtain:

$$\begin{array}{ccc} -M_{i}\leq & r_{j} & \leq 0\\ \epsilon_{i}\leq & \Delta G_{i} & \leq M_{i} \end{array}$$ (34)

That is, for negative flux rates, the corresponding Gibbs energy is positive. In case (ii) $z_{i}=1$ we obtain:

$$\begin{array}{ccc} 0\leq & r_{j} & \leq M_{i}\\ -M_{i}\leq & \Delta G_{i} & \leq -\epsilon_{i}z_{i} \end{array}$$ (35)

That is, for positive flux rates, the corresponding Gibbs energy is negative. This set of equation can now be written in matrix form in a very compact way. The full vector of variables that must be solved, now is as follows: $\left[\underline{r},\underline{z},\underline{\Delta G}\right]$ and the equation system with equality and inequality condition reads (sub-matrices $\hat{I}$ , I and 0, as well as M , and ϵ have appropriate dimensions):

$$\left(\begin{array}{ccc} -\hat{I} & M & 0\\ \hat{I} & -M & 0\\ 0 & -\left(M+\epsilon \right) & -I\\ 0 & \left(M+\epsilon \right) & I \end{array}\right)\left(\begin{array}{c} \underline{r}\\ \underline{z}\\ \underline{\Delta G} \end{array}\right)\leq \left(\begin{array}{c} M\\ 0\\ -\epsilon \\ M \end{array}\right),\left(\begin{array}{ccc} N & 0 & 0\\ 0 & 0 & K^{T} \end{array}\right)\left(\begin{array}{c} \underline{r}\\ \underline{z}\\ \underline{\Delta G} \end{array}\right)=\left(\begin{array}{c} 0\\ 0 \end{array}\right)$$ (36)

Note that matrix $\hat{I}$ is used to make clear that only internal reactions of the network are considered for the thermodynamic analysis, that is, all external rates have entry zero in $\hat{I}$ . Matrix K is the null space of the internal reactions as used above. Note, that cases with zero fluxes in the cycles might lead to inconsistencies because the corresponding ΔG value cannot be zero. However, in the Supplementary Appendix SA method is described that can be used for a verification, if a given flux map with zero entries in the internal flux vector is feasible.

A drawback of the procedure is, that additional variables including integer variables are needed that generate challenges while solving the equation system. An elegant way, described in (Beard et al., 2004), to avoid that fluxes are running in cycles is to formulate a non-linear equation based only on the sign of the fluxes (vector $\underline{s_{f}}$ ) and the sign of the cycles (vector $\underline{s_{c}}$ ) for $N^{\prime }$ :

$$|{\underline{s}}_{c}^{T}{\underline{s}}_{f}|<{\underline{s}}_{c}^{T}{\underline{s}}_{c}$$ (37)

Simply speaking, if a flux solution runs in a cycle, the left and the right hand side are equal; if this is not the case, then the left hand side is always smaller than the right hand side. Please note, however, that the strong constraint given in Equation 30 are not valid here; if there is potential between two components, a flux might be zero. In our case, for the sign vector we get ${\underline{s}}_{c}^{T}{\underline{s}}_{c}=3$ . In case that the flux distribution follows not the cycle, for example, ${\underline{s}}_{f}=\left[0;1;1;1\right]$ , we get $|{\underline{s}}_{c}^{T}{\underline{s}}_{f}|=1$ . In the normal case that the null space is high dimensional, the given equation should be applied to all cycles in the network. However, to determine the complete set of cycles is difficult, since linear combinations of null space vectors are also in the null space.

A further alternative, including the concentration of the metabolites and replacing the Gibbs energy by equations give above, allows us to find a further set of equations that could be used: Above, the condition $\Delta G_{i}r_{i}$ (Equation 21) was given that relates the sign of the Gibbs energy to the sign of the rate. Now this is applied to the cycle in the network. We restrict the analysis to a given flux vector, however, we only use the sign of the vector; $sign\left({\underline{r}}^{\prime }\right)$ . The set of equation read:

$$\left({\underline{\Delta G}}^{*0^{\prime }}+N^{\prime T}\ln\underline{x}\right)•sign\left({\underline{r}}^{\prime }\right)<0$$ (38)

Example 6

The set of equation is applied for the cycle in the network, and let us assume that $r_{2}$ and $r_{3}$ are positive and $r_{4}$ is negative, that is, fluxes are running in the cycle. Equation system (Equation 22) can be copied with slight modifications (we directly use the equilibrium constants and replace the equal signs with < and > , respectively):

$$\begin{array}{ccc} \Delta G_{2}^{*}<0\Rightarrow \ln\frac{c_{D}}{c_{B}^{2}K_{\mathrm{Eq2}}}<1\Rightarrow c_{D}<c_{B}^{2}K_{\mathrm{Eq2}} & & \\ \Delta G_{3}^{*}<0\Rightarrow \ln\frac{c_{B}}{c_{C}K_{\mathrm{Eq3}}}<1\Rightarrow c_{B}<c_{C}K_{\mathrm{Eq3}} & & \\ \Delta G_{4}^{*}>0\Rightarrow \ln\frac{c_{D}}{c_{C}^{2}K_{\mathrm{Eq4}}}>1\Rightarrow c_{D}>c_{C}^{2}K_{\mathrm{Eq4}} & & \end{array}$$ (39)

From the first two lines, it follows:

$$c_{D}<c_{B}^{2}K_{\mathrm{Eq2}}<{\left(c_{C}K_{\mathrm{Eq3}}\right)}^{2}K_{\mathrm{Eq2}}$$ (40)

with the condition for the equilibrium constants $K_{\mathrm{Eq2}}K_{\mathrm{Eq3}}^{2}=K_{\mathrm{Eq4}}$ , the last line from above reads:

$$c_{D}>c_{C}^{2}K_{\mathrm{Eq4}}=c_{C}^{2}K_{\mathrm{Eq3}}^{2}K_{\mathrm{Eq2}}$$ (41)

That is a contradiction to the first two lines; the flux cannot run in the cycle. The presented equations are sufficient for a loopless network.

Example 3c (re-opened)

**FIGURE 3:**
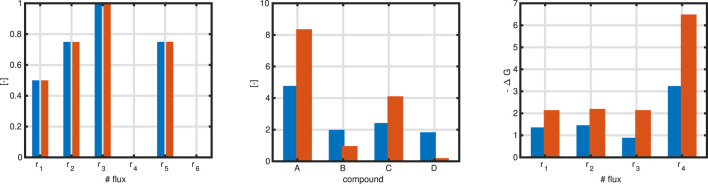
Left: Flux map with maximization of reaction $r_{3}$ under thermodynamic constraints (since the flux distribution in the Max-min case is given, the flux maps are the same). Middle: Bar plot for the concentrations of the compounds in the re-opened example 3c. Right: Bar plot for a comparison of ΔG values without and with the Max-min driving force approach. Blue bars for solutions of Equation 42, orange bars for solutions of Equation 43.

For the example, an extended optimization problem is formulated taking the concentrations of the compounds into consideration. The general form now reads:

$$\begin{array}{ccc} \max f:{\underline{c}}^{T}\underline{r} & = & c_{1}r_{1}+c_{2}r_{2}+\cdots c_{n}r_{n}\\ s.t.\;\; & & \\ N_{u}{\underline{r}}_{u} & = & -N_{b}{\underline{r}}_{b}\\ \left[{\underline{\Delta G}}^{*0^{\prime }}+N^{\prime T}\underline{x}\right]•sign\left({\underline{r}}^{\prime }\right) & < & \underline{0}\\ r_{i,min}\leq r_{i} & \leq & r_{i,max}\\ x_{j,min}\leq x_{j} & \leq & x_{j,max} \end{array}$$ (42)

with ${\underline{r}}^{\prime }$ takes into account only internal reaction rates and no exchange reactions. As above, algorithms used in the study require upper and lower bounds that are given in the last two lines. Example 3c is solved with equation system (Equation 42), and we get a different flux map fulfilling all constraints now (Figure 3).

However, there is a broad range for the values for the concentrations fulfilling the given constraints. In analogy of selection one extraordinary flux for the optimization procedure, we proceed the same way and select from all possible values for a set of concentrations one which fulfills additional specifications. As proposed in literature, for a given flux map, all (absolute) ΔG values for the internal reactions are determined (since the direction of a reaction is open, we are interested only in the quantity of ΔG ), and one tries to maximize the minimal value of these (Max-min driving force (MDF) design principle (Noor et al., 2014)). Formally, a different optimization problem is formulated based on given flux map ${\underline{r}}^{\prime }$ for the internal reactions only:

$$\begin{array}{ccc} \max f:\min|\Delta G_{i}| & & \\ s.t.\;\; & & \\ \left[{\underline{\Delta G}}^{*0^{\prime }}+N^{\prime T}\underline{x}\right]•sign\left({\underline{r}}^{\prime }\right) & < & \underline{0}\\ x_{j,min}\leq x_{j} & \leq & x_{j,max} \end{array}$$ (43)

Example 3c (continued)

Figure 3 left shows the updated and thermodynamically valid flux distribution. In the middle, shown in red, are the values for the concentration of the compounds without and with the MDF design principle. Right: The minimal value for the negative ΔG is higher in the MDF case. An interesting observation is that, although $\Delta G_{4}$ is higher than the other values, the corresponding flux $r_{4}$ is nearly zero. We note that, so far, fluxes and concentrations are only weekly coupled. Later on, this problem can be resolved by taking into account that fluxes are proportional to enzyme concentrations, and complete enzyme kinetics can be exploited.

**FIGURE 4:**
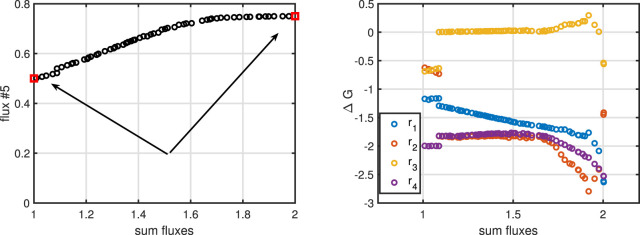
Left: Pareto front for maximizing flux $r_{5}$ and minimizing the sum of fluxes. With red symbols, the individual optima are shown (left minimum sum of fluxes, and right maximum $r_{5}$ ). The arrow points to the direction towards the Pareto front. All possible values are below the line that connects the two red symbols. Right: ΔG values for the four internal reactions over the minimum sum of fluxes.

To complete the example, we perform a multi objective approach to maximize flux $r_{5}$ , and minimize the sum of fluxes. The results of such a calculation can be plotted as a Pareto front. The values on the front are characterized by the following property: when we move along the front, only one of the two criteria is improved while the second one is impaired. For point that are not on the front, we can improve both criteria. The arrows in Figure 4 left indicates the direction for the improvement in both directions; crossing the front is not possible. Also, the ΔG values for the four internal reactions show a linear behavior.

Lessons learned from Example 3: To determine valid flux distributions for cellular reaction networks, mass conservation as well as thermodynamic principles must be considered. Depending on the choice of the objective function, flux maps may differ. The selection of the objective function depend on the experimental set-up. For growth of bacteria in surplus carbohydrate the maximization of the growth rate is an appropriate choice. However, in case that substrates are limited or for applications in metabolic engineering, different objective functions are more suitable.

### 2.3 Flux models with drain into biomass

**FIGURE 5:**
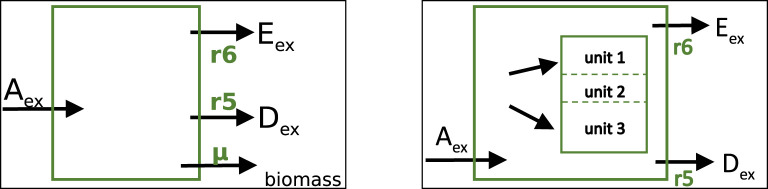
Left: Standard model representation in FBA with a single biomass flux as additional exchange reaction. Right: Model with several macromolecular units representing the major mass of a cell. Incoming and outgoing metabolic fluxes are the same as before and are the same in both approaches shown here.

In this section, the network is extended to take into account that metabolites in the network often serve as precursors for biomass synthesis. Two different variants will be considered as shown in Figure 5; first, the standard notation used in FBA is introduced, then, a modeling approach with several macromolecular units such as protein, RNA and DNA is considered.

A standard approach in FBA adds a single reaction, also called a pseudo-reaction, representing 1 g of biomass. In this single reaction, the complete metabolism for biomass assembly is summarized. For the example network from above, we use on a gram basis:

$$0.35\left(g\right)A+0.15\left(g\right)B+0.3\left(g\right)C+0.2\left(g\right)D\;\to \;1\left(g\right)biomass$$ (44)

The equation is converted with the molecular weight of the compounds and the stoichiometric matrix N has to extended with an additional column. On a molar basis, the equation reads in the FBA notation:

$$\text{2.33}\text{1}{\text{0}}^{-\text{3}}A+\text{1.5}\text{1}{\text{0}}^{-\text{3}}B+\text{3}\text{1}{\text{0}}^{-\text{3}}C+\text{1}{\text{0}}^{-\text{3}}D\overset{\mu }{\to }$$ (45)

In FBA, the right side in the last equation remains empty, that is, biomass is not explicitly taken into account as compound in the stoichiometric matrix and therefore, no additional row is necessary. This can also be seen in Figure 5 (right side); the reaction arrow points out of the system.

Aside note:

It is worth to have a closer look on the last equation since we changed the units from r given typically in [mmol/gDWh] to the growth rate μ , given in [1/h] . From our general approach for mass balance equations, we derived Equation 8; applying this to a compound (concentration is $c_{X}$ ) that is only synthesized in one reaction $r_{\mathrm{syn}}$ but not degraded (for examples macromolecules (with unit [mmol/gDW] ) or a representative for the overall biomass), one obtains:

$$0=r_{\mathrm{syn}}-\mu c_{X}\Rightarrow r_{\mathrm{syn}}=\mu c_{X}$$ (46)

Normally, as in the examples given in the beginning, the stoichiometric coefficients are dimensionless (essentially they are mol/mol ). For the special case of the biomass reaction, we write for example:

$$|\gamma_{A}|A+\cdots \overset{r_{\mathrm{syn}}=\mu c_{X}}{\to }\left(biomass\right)$$ (47)

but we do not consider compound *biomass* as an additional state variable. The o.d.e. for compound A

$$\dot{c_{A}}=\gamma_{A}r_{\mathrm{syn}}\cdots$$ (48)

is used and is now transformed by excluding the biomass concentration $c_{X}$ from the expression of $r_{\mathrm{syn}}$ and writing it directly to the stoichiometric coefficient:

$$|\gamma_{A}|\cdot c_{X}A+\cdots \overset{\mu }{\to }\;\left(biomass\right)\to \dot{c_{A}}=\left[\gamma_{A}\cdot c_{X}\right]\mu \cdots$$ (49)

The last equation is re-written:

$$|\gamma_{A}^{\prime }|A+\cdots \overset{\mu }{\to }\;\left(biomass\right)\to \dot{c_{A}}=\gamma_{A}^{\prime }\mu \cdots$$ (50)

We note a change of units in the stoichiometric coefficients; $\gamma_{A}$ is mol/mol that is now changed for $\gamma_{A}^{\prime }$ in mol/mol⋅mol/gDW=mol/gDW . If, like in our case, the reaction equation describes the synthesis of the overall biomass, the rate of synthesis can simply be replaced by the growth rate μ , and the stoichiometric coefficients with unit [mol/gDW] represent with biomass scaled values.

In Example 7 the drain of metabolites to generate biomass is considered and as in the previous case, two different objective functions are compared. In addition, constraints by the available amount of enzyme for the network is studied, resulting in four different scenarios.

Example 7

**FIGURE 6:**
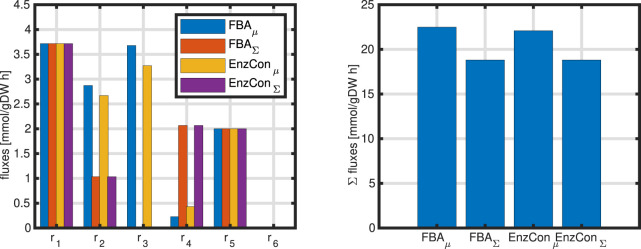
Left: Comparison of simulation results for four different cases; standard FBA (two objectives: maximizing growth rate and minimizing sum of fluxes) and enzyme constraint (two objectives: maximizing growth rate and minimizing sum of enzymes). The values for the incoming fluxes and for the growth rate are equal for all cases $r_{in}=10mmol/gDWh,\mu =1.1$ 1/*h*. Right: Sum of all fluxes for the four different cases.

The network considered so far is extended by a single biomass reaction (Equation 44). Simulations results are shown in Figure 6 with two cases: (i) maximization of growth rate, and (ii) minimization of the sum of all fluxes.

For the next level, a further extension of the stoichiometric/thermodynamic approach is introduced. It becomes obvious during the last decade, that the standard approach has a strong disadvantage; the biomass constituents are not considered, and therefore, any feedback from the biomass parts back to the metabolic network is missing. This is true especially for proteins like enzymes that are involved in any metabolic processes. Measurement of the proteome now offers several possibilities to extend the flux analysis approach by taking into account not only different protein classes or sectors, but also information on individual enzymes. In a step-by-step approach we provide, and also combine, several methods from literature. The intention is to show that protein allocation has a strong impact on flux distributions, which has implications for the understanding of cellular physiology and but also is important for applications in Metabolic Engineering where one is interested to redirect fluxes to desired products.

Considering individual enzymes, from kinetics, it is convenient to split the mathematical term into two parts in a first step. Such rate laws depend on the concentration of all involved substrates S , products P , and the overall amount of enzyme $E_{0}$ in the reaction assay. If the enzyme is under control by inhibition or activation, also the concentration of the effectors come into play. The kinetics can be written in the following form with a set of parameter $\underline{p}$ :

$$r=f^{\prime }\left(S,P,E_{0},\underline{p}\right)=k_{\mathrm{cat}}E_{0}f\left(S,P,\underline{p}\right)$$ (51)

From here we see, that $r<k_{\mathrm{cat}}E_{0}$ because for the term f , the following relation holds: 0≤f≤1 for a irreversibel reaction. Moreover, it is observed that the total amount of protein during different conditions does not change too much, indicating that the sum over all enzymes $\sum E_{0i}$ could (i) be constraint, or could (ii) be subject of optimization, since a minimal sum of metabolic enzyme could allow the cell to spend more proteins in ribosomes to accelerate growth. Both ideas ((i) and (ii)) are described in literature as GECKO (Sanchez et al., 2017) (we used a slightly modified version here) or ECM [enzyme cost minimization (Noor et al., 2016)].

Taking into account additional variable for the single enzyme $E_{i}$ (for simplicity we omit the zero for the total amount of this enzyme), Equation system (Equation 11) is complemented. With the same objective function as before, one gets for case (i):

$$\begin{array}{ccc} \max f\left(\text{or}\min f\right):{\underline{c}}^{T}\underline{r} & = & c_{1}r_{1}+c_{2}r_{2}+\cdots c_{n}r_{n}\\ s.t. & & \\ N_{u}{\underline{r}}_{u} & = & -N_{k}{\underline{r}}_{k}\\ I_{u,u}|\underline{r_{u}}|-K_{c}{\underline{E}}_{i} & < & \underline{0}\\ \sum E_{i} & < & E\\ 0\leq E_{i} & \leq & E_{i,max}\\ r_{i,min}\leq r_{i} & \leq & r_{i,max} \end{array}$$ (52)

with $K_{c}$ is a diagonal matrix with values $k_{\mathrm{cat}}$ , corresponding to the unknown rates $r_{u,i}$ ; E is a parameter that limits the total amount of enzyme that is available for the network. Since the direction of the fluxes is open we must use the absolute values of the rates here.

In case (ii) a different objective function is used; namely, the sum of all enzymes in the network is minimized. Here, the equation system reads with weighting factors, for example the molecular weight:

$$\begin{array}{ccc} \min f:\sum w_{i}E_{i} & = & w_{1}E_{1}+w_{2}E_{2}+\cdots w_{n}E_{n}\\ s.t. & & \\ N_{u}{\underline{r}}_{u} & = & -N_{k}{\underline{r}}_{k}\\ I_{u,u}|\underline{r_{u}}|-K_{c}{\underline{E}}_{i} & < & \underline{0}\\ \sum E_{i} & < & E\\ 0\leq E_{i} & \leq & E_{i,max}\\ r_{i,min}\leq r_{i} & \leq & r_{i,max} \end{array}$$ (53)

In case that only positive fluxes are considered (this can be achieved by splitting reversible fluxes into two positive ones), the two inequalities can be re-written in matrix notation with matrices/vectors with apparent size:

$$\left(\begin{array}{cc} I & -K_{c}\\ \underline{0} & \underline{1} \end{array}\right)<\left(\begin{array}{c} \underline{0}\\ E \end{array}\right)$$ (54)

Example 8

For the given network, a rough estimation of the $k_{\mathrm{cat}}$ can be done. From published proteome data, a mean value of E=2000 transporter molecules per cell is obtained. For a calculation with a standard uptake rate in the range of r=6mmol/gDWh , the number of molecules per cell must be transformed into the unit mol/gDW . Here, we need Avogadro’s number Av and an average cell mass $m_{c}$ $\left(\text{1}\text{1}{\text{0}}^{-\text{12}}gDW\right)$ :

$$c_{E}=\frac{E}{Avm_{c}}=\text{3,3}\text{1}{\text{0}}^{-\text{3}}\mu mol/gDW$$ (55)

and one obtains for the $k_{\mathrm{cat}}$ :

$$k_{\mathrm{cat}}=\frac{r}{c_{E}}=\text{1,8}\text{1}{\text{0}}^{\text{6}}\text{1}/h$$ (56)

Example 7 (continued)

Results of the different simulation studies introduced are shown in Figure 6. For a fair comparison, the growth rate was fixed in case of minimizing the sum of fluxes or minimizing the sum of enzymes to the same value that was obtained while maximizing the growth rate. The weights $w_{j}$ from above are taken as one. As can be seen, depending on the objective function, the flux distribution changes for nearly all rates ( $r_{5}$ and $r_{6}$ were set before and do also not change). The right plot in the Figure shows the sum of all fluxes. As expected the second objective function results in lower values, but the difference is only about ≈ 16% with respect to the first objective.

Lessons learned from Example 7: The choice of the objective function and additional constraints show a strong impact on the flux distribution. However, while some fluxes does not change (for example $r_{1}$ , $r_{5}$ , and $r_{6}$ ) other fluxes show drastic changes.

### 2.4 Flux models with several macromolecular units

The approaches so far still have a couple of limitations. The results are scalable since, typically, the main incoming fluxes must be given, and cannot get as a result from the optimization procedure; second, the biomass equation is an approximation, and does not account for different needs of the cell for growth and maintenance. In the next step, we omit the single biomass reaction and replace it with macromolecular units that are not subject to degradation (see Figure 5 right).

From the basic mass balance Equation 8, a steady state can only be reached, if the dilution term is considered. Also, to keep our mass conservative approach alive, the growth rate must be the result of incoming mass flow minus outgoing mass flow. Fortunately, from the basic equation, a simple, but powerful relationship could be derived for the specific growth rate (Doan et al., 2022):

$$\mu =\underline{w}N\underline{r}$$ (57)

The multiplication of N from the left side with the molecular masses always results in entries 0 when the respective reaction is completely mass conservative. All entries that are not zero contribute either positively or negatively to the specific growth rate μ . The equation can directly plugged in our standard equation, and one obtains an extended steady state condition:

$$\left(I-{\underline{w}}^{T}\underline{c}\right)N\underline{r}=MN\underline{r}=\underline{0}$$ (58)

In comparison with standard FBA, an additional matrix M comes into play that takes into account the concentration of all compounds and the macromolecular units. Fortunately, the system is still linear (if thermodynamics is not considered) in the fluxes and can be solved with standard tools. In addition to the flux distribution that were obtained so far, additional solutions appear that describe fluxes into the biomass. However, a close inspection of the equation reveals that the composition of the cell must be known (represented in vector $\underline{c}$ ). Rough values for the macromolecular composition can be found in literature, but concentrations values for small molecules are difficult to obtain. However, for first calculations, we can set these values to zero.

In Example 9, a simple model structure is used to analyze the influence of the biomass macromolecular structure. It is assumed that these elements are not actively degraded, and therefore only diluted by biomass growth. This allows model reduction, and, as shown in the extended case, also allows to study the influence of feedback structures, that is, the macromolecules influence their own synthesis.

Example 9

A simple scheme with one uptake reaction, one reaction that excreeds the metabolite directly back into the medium (this is based on experimental observations and is termed “overflow”^5^), and two cellular units $B_{1}$ and $B_{2}$ is considered. Metabolite A , a catabolic product, represents a compound from central metabolism or an amino acid that is needed to build macromolecular units like protein, lipids, or DNA.

**FIGURE 7:**
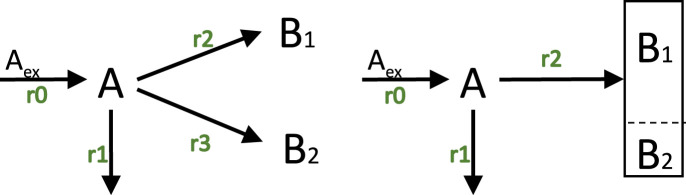
Left: Network with three unknown fluxes $\left(r_{1},r_{2},r_{3}\right)$ and two macromolecular units. Right: Reduced model from the left side. Incoming and outgoing metabolic fluxes are the same in both approaches shown here.

The stoichiometric matrix N for the example shown in Figure 7, left, reads as follows, taking into account that a higher number of monomers are needed for 1 mol of a macromolecular unit (that is, $\gamma_{2}$ and $\gamma_{3}$ have large values):

$$N=\left(\begin{array}{cccc} 1 & -1 & -\gamma_{2} & -\gamma_{3}\\ 0 & 0 & 1 & 0\\ 0 & 0 & 0 & 1 \end{array}\right);$$ (59)

with the first column represents the input flux as before. The molecular weights are in this case $w_{A},w_{B1}$ and $w_{B2}$ . However, the quantities are not independent; for example, we get for the vector with the molecular weights:

$$\underline{w}=\left(\begin{array}{c} w_{A}\\ \gamma_{2}w_{A}\\ \gamma_{3}w_{A} \end{array}\right)$$ (60)

While the masses of the compounds only fulfill the conservation condition (to sum up to 1g biomass). A closer look at the o.d.e’s for the biomass components reveals a further interesting property that can be used for the numerical solution. For macromolecular units $B_{1}$ and $B_{2}$ , the respective equations read:

$$\begin{array}{ccc} {\dot{B}}_{1}=r_{2}-\mu B_{1} & & \\ {\dot{B}}_{2}=r_{3}-\mu B_{2} & & \end{array}$$ (61)

One gets for the steady state:

$$\begin{array}{ccc} r_{2}=\mu B_{1}\to \frac{r_{2}}{B_{1}}=\mu & & \\ r_{3}=\mu B_{2}\to \frac{r_{3}}{B_{2}}=\mu & & \end{array}$$ (62)

The nice thing with the last equations is, that scaling of the rate of synthesis with the molecular mass fractions always result in the specific growth rate, that is, the rates of synthesis are not independent (here: $r_{3}=r_{2}\frac{B_{2}}{B_{1}}$ ). Vice versa, knowing the specific growth rate, that in the example can easily be determined:

$$\mu =w_{A}\left(r_{0}-r_{1}\right)$$ (63)

The rates of synthesis for the macromolecular units can be obtained, and does not require the solving of an additional equation system. For this model, the null space K can be used to determine all solutions of the system, and for the example, we obtain for the special case that the mass fraction of compound A is negligible $f_{A}=0$ :

$$K=\left(\begin{array}{cc} 1 & \gamma_{3}/\left(1-f_{B1}\right)\\ 1 & 0\\ 0 & \left(f_{B1}\gamma_{3}\right)/\left(\gamma_{2}\left(1-f_{B1}\right)\right)\\ 0 & 1 \end{array}\right)$$ (64)

The first null space vector shows the way of the substrate through the network into the excreted overflow product (column 1) while the second vector represents the growth mode of the network (column 2), that is, the way from the substrate into the macromolecular units. A close inspection of the third element in the second vectors reveals that the element can be rewritten as $k_{32}=B_{1}/B_{2}$ , that confirms the statement from above.

As indicated in Figure 7a reduced model can be obtained with only one single flux into the structured biomass (2 fractions). In the reduced model, the biomass is structured in two parts with only one rate.

Aside note:

If the stoichiometric N is re-written for the two biomass reactions in such a way that the mass composition is directly included, then the null space directly shows that all rates of biomass (or macromolecular) synthesis are equal to one for the growth mode and for the assumption $f_{A}=0$ . For the example above, N then reads:

$$N=\left(\begin{array}{c} 1-1-f_{B1}/w_{A}-\left.\left(1-f_{B1}\right)/w_{A}\right)\\ 0\;\;\;0f_{B1}/w_{B1}0\\ 000\left.\left(1-f_{B1}\right)/w_{B2}\right) \end{array}\right)$$ (65)

and the product M⋅N is:

$$MN=\left(\begin{array}{cccc} 1 & -1 & -f_{B1}/w_{A} & -\left(1-f_{B1}\right)/w_{A}\\ -f_{B1}/\gamma_{2} & f_{B1}/\gamma_{2} & f_{B1}/\left(\gamma_{2}w_{A}\right) & 0\\ -\left(1-f_{B1}\right)/\gamma_{3} & \left(1-f_{B1}\right)/\gamma_{3} & 0 & \left(1-f_{B1}\right)/\left(\gamma_{3}w_{A}\right) \end{array}\right)$$ (66)

and the null space K is:

$$K=\left(\begin{array}{cc} 1 & 1/w_{A}\\ 1 & 0\\ 0 & 1\\ 0 & 1 \end{array}\right)$$ (67)

Example 9 (continued)

**FIGURE 8:**
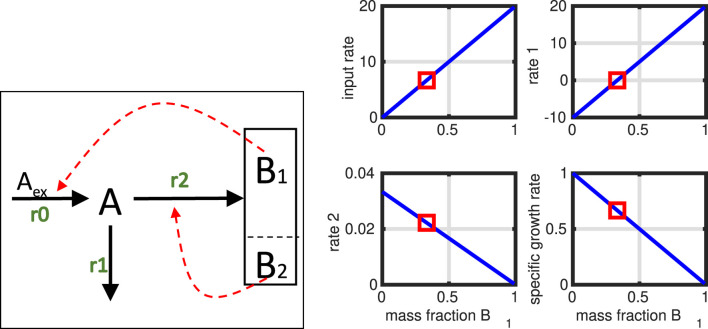
Controlled network; in a feedback loop, the macromolecular units directly influence the fluxes in network. Unit $B_{1}$ has a feedback effect on the incoming transport flux while unit $B_{2}$ has a feedback effect on the rate of its own synthesis (red arrows). Right: Complete solution space as a function of $B_{1}$ (all rates and specific growth rate) with optimal growth rate (red square) indicated directly in the plot.

Here, the reduced model is considered and a feedback structure is introduced as shown in Figure 8; both fractions of the biomass are needed to sustain the incoming flux $r_{1}$ (fraction $B_{1}$ ) and drain to biomass flux $r_{2}$ (fraction $B_{2}$ ). Here, we note, that the allocation of both fractions play an important role for the system. Depending on the incoming flux, the biomass must be distributed in such a way that both rates can sustain their fluxes. For a first simulation, the biomass composition is varied and the concentration of metabolite A is set to zero. Since the total biomass is scaled to 1g , only one control variable (here fraction $B_{1}$ ) is needed to explore the complete solution space shown in Figure 8 right.

The equation system starts from the standard equation system (Equation 58). Now, the rate vector (or parts of it) is replaced by a simple kinetic expression $r∼B_{j}$ , that is in our case, $r_{0}=k_{1}B_{1}$ , and $r_{2}=k_{2}B_{2}$ (in this way, the system is still linear):

$$\underline{0}=\left(I-{\underline{w}}^{T}\underline{c}\right)N\underline{r}=MN\left(\begin{array}{ccc} k_{1} & 0 & 0\\ 0 & 1 & 0\\ 0 & 0 & k_{2} \end{array}\right)\left(\begin{array}{c} B_{1}\\ r_{1}\\ B_{2} \end{array}\right);$$ (68)

However, we must add the conservation equation, saying that the sum of $B_{1}$ and $B_{2}$ is constant and represents the overall biomass concentration B :

$$B=\left(\begin{array}{ccc} 1 & 0 & 1 \end{array}\right)\left(\begin{array}{c} B_{1}\\ r_{1}\\ B_{2} \end{array}\right)$$ (69)

and we optimize for the specific growth rate $\mu =w_{A}\left(r_{0}-r_{1}\right)$ .

Figure 8 shows the network structure and the simulation results. Since only only degree of freedom exists, the complete (linear) solution space could be determined (incoming flux $r_{0}$ , $r_{1}$ , and $r_{2}$ as well as the specific growth rate in dependence on fraction $B_{1}$ ). However, the results are not intuitive. The optimal value for the growth rate is shown with a red square. Smaller values than the optimal one for $B_{1}$ (values on the left side) result in a higher growth rate that could not be realized by the incoming flux (note that $r_{1}$ is negative). Higher values of $B_{1}$ (values on the right side) lead to a lower growth rate because $B_{1}$ is too high and fraction $B_{2}$ , responsible for its own generation, is too small. With decreasing rate $r_{2}$ , rate $r_{1}$ must increase further to reach a steady state.

**FIGURE 9:**
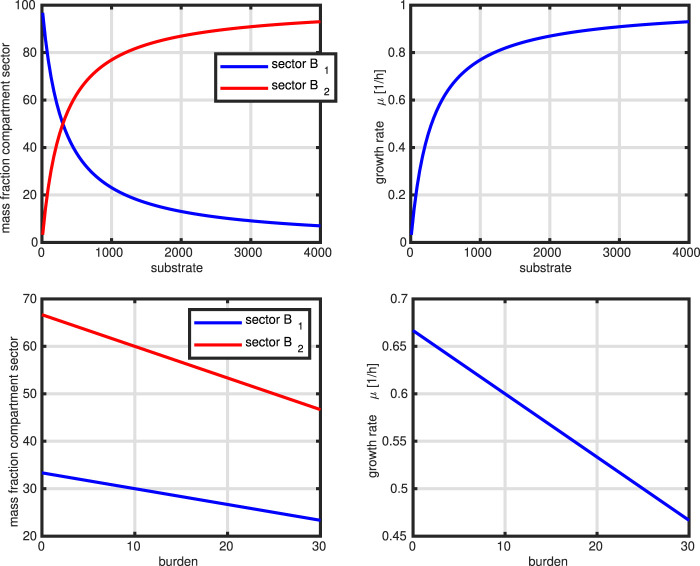
Controlled network. Upper row: mimic of substrate limitation (course of the two sectors, course of the growth rate) over the substrate concentration. Lower row: Mimic of heterologous protein production (course of the two sectors, course of the growth rate) over metabolic burden.

In the next Figure 9, we show two simulation studies with this simple model. First, we mimic a limitation of substrate in the medium (two left plots). This is realized by changing parameter $k_{1}$ and the x-axis in this case represents the limiting substrate concentration (arbitrary units). The plots on the right side show a simulation study when we assume that not all B is available for $B_{1}$ or $B_{2}$ . This case mimics for example, the synthesis of a heterologous protein.

The examples already demonstrate a self contained model system. In comparison to the previous models, substrate intake is limited by the available amount on sector $B_{1}$ . Actually, increasing the input flux is possible by shifting the resources to $B_{1}$ , however, of costs of synthesis of biomass. The optimal growth rate is a compromise between substrate uptake and biomass synthesis that, of course, depends on the kinetic and stoichiometric parameters used. The model also reproduces the behavior observed for protein sectors in *Escherichia coli* in the two applications. Studies on proteome data for different substrates and growth conditions reveal that the fraction for carbohydrate transport and central pathways is decreasing with the growth rate while the ribosomal fraction is increasing Schmidt et al. (2016). For this case, one also could expect a different behavior of the system with respect to resource allocation. The optimal solution indicates that the loss of resources due to the additional protein production is equally distributed to both fractions, however, a different solution could be that one sector stays constant while the load is given to only the second sector. One interesting observation is, that in contrast to literature, the behavior of the growth rate in the first application is not linear but shows a hyperbolic outcome and approaches a threshold. This is understandable since - although substrate uptake is linear and not saturated–the resources can be distributed either to the incoming uptake unit or to the synthesis unit.

The last set of examples introduces a new aspect in modeling cellular systems, namely, the dependencies of the reaction rate from the concentration of substrates, products and parameters. The analysis start with the network from Example 3 and then extends it with kinetics. The final case takes into account that the resources to generate the biomass macromolecular structure is limited by the proteins. In this way, a self contained cellular model is obtained.

Example 10a

**FIGURE 10:**
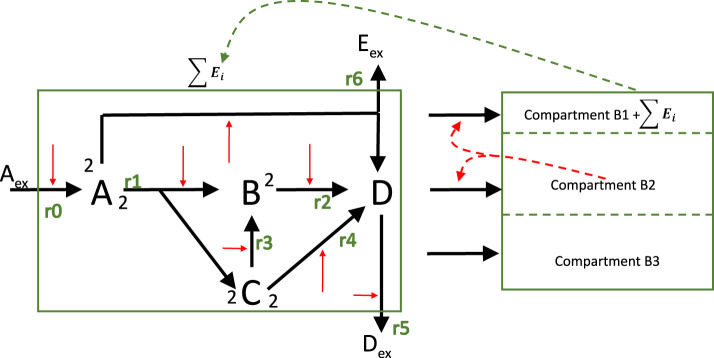
Overall structure of the model. The stoichiometric network is combined with the macromolecular unit modeling approach. For the model with resource allocation, feedback control is realized by using part of $B_{1}$ as enzymes for the metabolic part and $B_{2}$ in charge to sustain B1+∑E and $B_{2}$ itself; unit $B_{3}$ is inert. All reactions in the network are catalyzed by enzymes (red arrows) and enzyme kinetics are used as rate laws.

In the next step, our example with 4 metabolites is considered again and extended with three macromolecular units. In Figure 10 the structure of model variant is shown that is used for three different cases.

In the first variant, the macromolecular units are fixed and the stoichiometric matrix contains the drain from the metabolites into the respective units. It is assumed that the sum of the mass fraction of the metabolites ( A , B , C and D ) is 0,1g/g that is 10% of the entire mass. This variant is yet not self containing; therefore the input flux must be fixed. Here, two objectives are considered: maximizing growth rate and minimizing the sum of fluxes (by a given input flux). Results are shown later on and are compared with the other cases (see Figure 12).

### 2.5 Kinetics–the step to dynamic models

Now we go back to Equation 51 and fill the kinetics with “life”, that is, we consider the dependencies of rates $r_{j}$ not only from enzymes, but also from the concentration of reaction partners. In literature, numerous kinetic expression can be found taking into account the number of catalytic centers as well as the number of substrates and/or products. The best known kinetics is the Michaelis-Menten (MM) kinetics that reads:

$$\text{MM kinetics}\;r=k_{\mathrm{cat}}E\frac{S}{S+K_{M}}$$ (70)

with the substrate concentration S and the half saturation parameter $K_{M}$ (also called Michaels-Menten constant). This kinetic describes the irreversible conversion of one molecule of substrate S into one molecule P . To be more flexible, for the following studies, a reversible reaction is used here, that allow a general conversion of $n_{i}$ molecules of $S_{i}$ into a number $n_{j}$ molecules of products $P_{j}$ (Wortel et al., 2014):

$$r=k_{\mathrm{cat}}E\frac{\prod {\left(S_{i}/K_{s}\right)}^{n_{i}}\left(1-\frac{\prod \overset{n_{j}}{\underset{j}{P}}}{\prod \overset{n_{i}}{\underset{i}{S}}K_{Eq}}\right)}{\prod {\left(1+s_{i}\right)}^{n_{i}}+\prod {\left(1+p_{j}\right)}^{n_{j}}-1}$$ (71)

with the abbreviations $s=S/K_{s}$ and $p=PK_{p}$ in the denominator; $K_{s}$ and $K_{p}$ are the respective binding constants for substrate and product. The equation can be structured into three parts: one capacity term $\eta_{c}$ indicating the maximal value of the velocity, a saturation term $\eta_{s}$ between zero and one, taking into account the number and type of binding centers for substrate, product and possible effectors (not considered here), and finally, a thermodynamic term $\eta_{t}$ that describes the capability of reaction reversibility (Noor et al., 2013):

$$\begin{array}{ccc} \eta_{c} & = & k_{\mathrm{cat}}E\\ \eta_{s} & = & \frac{\prod {\left(S_{i}/K_{s}\right)}^{n_{i}}}{\prod {\left(1+s_{i}\right)}^{n_{i}}+\prod {\left(1+p_{j}\right)}^{n_{j}}-1}\\ \eta_{t} & = & 1-\frac{\prod \overset{n_{j}}{\underset{j}{P}}}{\prod \overset{n_{i}}{\underset{i}{S}}K_{Eq}}=1-e^{\Delta G^{*}} \end{array}$$ (72)

As seen above for a simple kinetic, a relationship in case of a reaction equilibrium could be obtained by setting $r=\eta_{t}=0$ . Here we obtain:

$$1-\frac{\prod \overset{n_{j}}{\underset{j}{P}}}{\prod \overset{n_{i}}{\underset{i}{S}}K_{Eq}}=0$$ (73)

and the equilibrium constant is obtained like before:

$$K_{Eq}=\frac{\prod \overset{n_{j}}{\underset{j}{P}}}{\prod \overset{n_{i}}{\underset{i}{S}}}$$ (74)

Please note, that in this framework, the value of $\Delta G^{*}$ determines the sign of the reaction (for example, if $\Delta G^{*}<0$ , the reaction rate r is always positive).

Aside note:

In reversible enzyme kinetics, the equilibrium constant $K_{eq}$ plays an important role and determines not only the direction of the reaction, but also the Gibbs energy. The reaction kinetics for a simple reversible reactions reads for 1A→1B :

$$r=k_{\mathrm{cat}}E\frac{S/K_{S}\left(1-\frac{P}{SK_{Eq}}\right)}{\left(1+s/K_{s}+PK_{p}\right.}$$ (75)

We note that the kinetic parameters of substrate binding $\left(K_{S}\right)$ and product binding $\left(K_{p}\right)$ as well as the reactions parameter for the forward and the backward reaction $k_{\mathrm{cat}}^{+}$ and $k_{\mathrm{cat}}^{-}$ , all contribute to the overall equilibrium constant $K_{Eq}$ ; this relationship is called the Haldane relationship and can be written:

$$K_{Eq}=\frac{k_{\mathrm{cat}}^{+}K_{P}}{k_{\mathrm{cat}}^{-}K_{S}}$$ (76)

**FIGURE 11:**
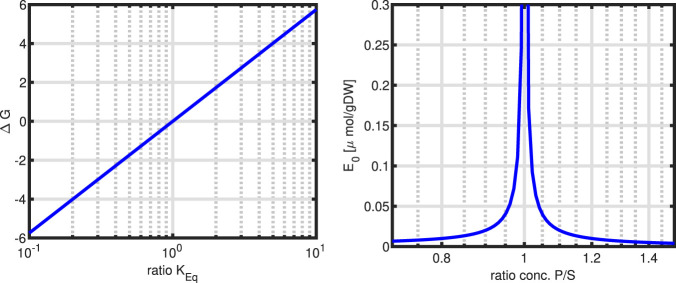
Left: Change of ΔG as a function of parameter $K_{Eq}$ . Right: Amount of enzyme needed to take a flux of r=1mmol/gDWh with $k_{\mathrm{cat}}=10^{6}$ for varying product P/S ( S is constant), $K_{Eq}=1$ and constant $S/K_{s}=1$ . Note that a value of 1 on the x-axis is the reaction equilibrium (r=0) and the amount of enzyme goes to infinity.

Keeping S and P constant and varying $K_{Eq}$ in the order of one magnitude, the Gibbs energy changes 5,7kJ/mol (left plot in Figure 11, R=8,315J/molK and T=300K ). This can be seen by a simple calculation; from the expression for ΔG , the difference for two values of $K_{Eq}$ with a factor of 10 is taken:

$$\begin{array}{ccc} \Delta G_{1}-\Delta G_{2} & = & RT\left(\ln\frac{P}{SK_{\mathrm{Eq1}}}-\ln\frac{P}{SK_{\mathrm{Eq2}}}\right)\\ & = & RT\ln\frac{K_{\mathrm{Eq2}}}{K_{\mathrm{Eq1}}}=2,5kJ/mol\ln10=5,75kJ/mol \end{array}$$ (77)

However, taking Equation 75 to determine the amount of enzyme needed for varying product, a highly sensitive behaviour is observed (right plot in Figure 11). Near the equilibrium constant, the amount of enzyme needed to maintain a flux of r=1mmol/gDW the amount of enzymes raises very fast.

Furthermore, the above considerations on thermodynamic properties in cycles are taken into account, that is, the equilibrium constants are not independent, but must fulfill the condition given in Equation 24.

Example 10b (now with kinetics)

**FIGURE 12:**
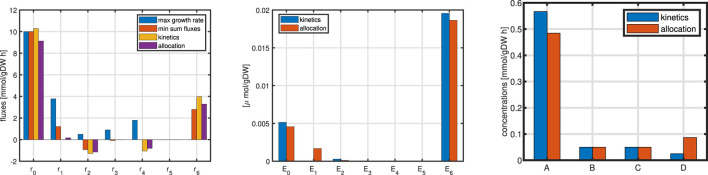
Left: Flux distribution for all rates for all cases. Middle: Comparison of enzyme concentrations for cases 10b and 10c. Right: metabolite concentrations for cases 10b and 10c.

Now the same network as in Example 10a is considered, but kinetics for the metabolic network are taken into account. The system is now self-contained with a minimal number of constraints: mass fraction of metabolites is again fixed and the concentration of the unitss are given. For the models considered so far, the concentration of metabolites only had a modest role; they were only important for thermodynamic considerations. Figure 12 shows the results. Please note that for the equation for one macromolecular unit, the following equation holds in steady state:

$$r_{\mathrm{syn,j}}=\mu B_{j}$$ (78)

Since in this case, the concentration of the unit is fixed and μ is only calculated via the metabolic network, the rate of synthesis $r_{\mathrm{syn}}$ can be directly obtained from the equation, and no further dependencies, for example from the metabolites, are necessary.

Example 10c (resource limitation)

In the last case, the first unit $B_{1}$ is responsible for all enzymes that are active in the metabolic network, that is $\sum E_{i}$ contributes to $B_{1}$ (there is a fixed amount that is increased by the sum of enzymes). Unit $B_{2}$ is in charge for the synthesis of B1+∑E and for its own synthesis. Unit $B_{3}$ is is obtained by closing the mass balance, accordingly, its rate of synthesis can be determined from its concentration. Still, the sum of the metabolites corresponds to 10% of the entire cell mass (a minimum concentration is also required, that is, the concentration of a metabolite cannot be zero). Figure 12 compares results for three cases. For the equation for a self controlled unit (example 10c) a different equation holds in steady state:

$$r_{syn_{j}}\left(B_{j}\right)=\mu B_{j}$$ (79)

A simple first order dependency in the form $k_{\mathrm{syn,j}}B_{j}$ would immediately result in $\mu =k_{\mathrm{syn,j}}$ and $B_{j}$ arbitrary, which is independent from the complete network and from the environmental conditions. Since this is not useful, a simple kinetics, coupling the metabolic network to the synthesis unit, is used here:

$$r_{syn_{j}}\left(B_{j},M\right)=k_{\mathrm{syn,j}}f\left(M\right)B_{j}\;\text{with for example}\;f\left(M\right)=\frac{M}{K_{M}}$$ (80)

The optimization problem is now as follows (for all cases in Example 10):

$$\begin{array}{ccc} \max f: & = & \mu ={\underline{w}}^{T}N\underline{r}\;\text{example }10\text{a,b,c}\\ \min f: & = & \sum r_{i}\;\text{example }10\text{a}\\ s.t. & & \\ MN\underline{r} & = & 0\;\text{example }10\text{a, no kinetics}\\ MN\underline{r}\left(\underline{E},\underline{C}\right) & = & 0\;10\text{b,c with kinetics}\\ \sum E_{i} & \leq & E\;\text{sum of enzyme is constraint, }10\text{b,c}\\ \sum C_{i} & = & C\;\text{sum of metabolites is constraint, }10\text{b,c}\\ 0\leq E_{i} & \leq & E_{i,max}\\ c_{i,min}\leq c_{i} & \leq & c_{i,max}\\ C+B_{1}+E_{\mathrm{mass}}+B_{2}+B_{3} & = & 1\;\text{total mass is constraint} \end{array}$$ (81)

The substrate uptake rates in all cases are comparable (left plot) while the flux maps differ: rate $r_{6}$ is used in all but one case while rates $r_{2-4}$ are used in the positive direction only in the first case. For example 10b and c, enzyme is allocated for the uptake rate and for the synthesis of metabolite D which fill up all other metabolites in the reversed direction (rates $r_{2-4}$ are negative).

The last model variant finally is used to mimic the behavior for a decreasing substrate concentration. In the previous example, the enzyme for substrate uptake is working in saturation, that is, the concentration of the substrate S is far larger than the affinity constant and the flux can be approximated by a linear relationship $r=k_{\mathrm{cat}}ES$ . Now, the substrate concentration is changed and the results are plotted over the concentration of S .

Although the input flux is linear, the resulting growth rate tends to saturate for high values. This is based on (i) the saturation kinetics of the individual enzymes in the network, and (ii) on the limited capacity of the enzyme fraction that can be allocated for substrate uptake; please note, that for the synthesis of enzymes (and unit $B_{1}$ ), unit $B_{2}$ must be synthesized too. Therefore, as can be seen, a typical behavior for the course of the units can be detected; unit $B_{2}$ which is self-controlled increases with the substrate (and with growth rate μ ) while unit $B_{3}$ (remainder) decreases. The amount of enzyme needed is included in $B_{1}$ but is not visible in the simulation. For high values of the substrate, only the enzyme for substrate uptake $\left(E_{0}\right)$ and $E_{6}$ contributes significantly; for smaller values, the pattern changes, and enzyme $E_{1}$ is needed to maintain the flux from A to B and C which was not necessary before. Interestingly, the yield (that is biomass generated from substrate) increases for small values of S ; this is due the lower flux via $r_{6}$ that besides D generates also a by-product (see Figure 10).

**FIGURE 13:**
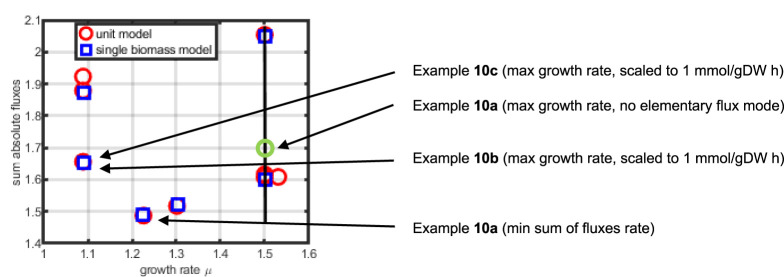
Sum of fluxes as a function of the growth rate for elementary flux modes (red circles indicate the elementary flux modes for the three unit model, blue squares for the single biomass equation model). Shown are the results for example 10.

We finally compare and illustrate the results of the simulation studies with biomass using elementary flux modes (see Supplementary Appendix for a definition and an example). For both networks, elementary flux modes with substrate uptake (from $A_{ex}$ ) and building biomass are considered. Figure 13 shows the relationship between the obtained growth rate and the sum of fluxes (only metabolic fluxes). The black line indicates the maximal growth rate that can be obtained from substrate uptake of S of 10mmol/gDWh .

As can be seen, the macromolecular unit model has more elementary flux modes than the single biomass model, showing a higher flexibility in providing precursors for biomass. However, the elementary flux mode with a higher growth rate than μ=1,5 (the red symbol on the right side of the black line) includes an uptake of a second substrate (that is, the corresponding product is not build but serves as a substrate). As expected, while minimizing the sum of all fluxes, an elementary flux mode is directly obtained, the values obtained from example 10b and 10c (only growth rate maximization) are only near an elementary flux mode but do hit them perfectly.

**FIGURE 14:**
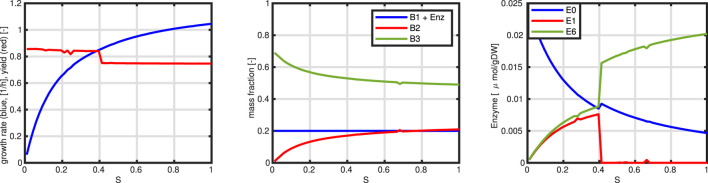
Left: Growth rate μ (blue) and yield (red). Middle: Course of the macromolecular units over substrate concentration (the sum of all unit fraction add to 1g biomass). Right: Course of enzymes for uptake rate and rates $r_{1}$ and $r_{6}$ .

A further analysis can be done by focusing on one node in the network and characterizing the incoming fluxes, that are named supply fluxes, and the outgoing fluxes, named demand fluxes. In steady-state, supply and demand are exactly balanced, that is, the sum of supply fluxes must match the demand fluxes. Moreover, since kinetic expressions are used for the last examples, the influence of metabolic concentrations can be studied. In the last example shown in Figure 14, the distribution of enzyme over the substrate input abruptly changes. In the following figure, two values of substrate input, corresponding to growth rates μ=1,011/h (high) and μ=0,651/h (low) are compared.

**FIGURE 15:**
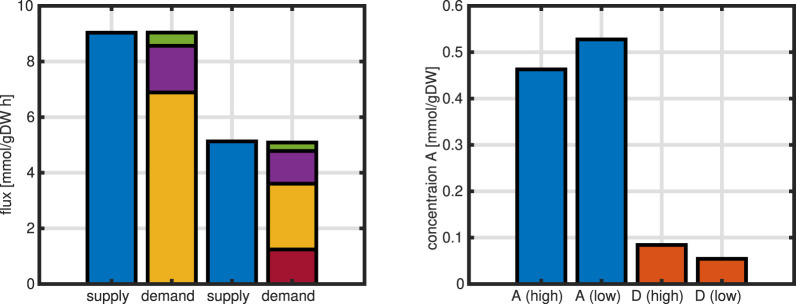
Left: first 2 bars are supply (left) and demand (right) for the high growth rate for metabolite A , remaining 2 bars are supply (left) and demand (right) for the low growth rate for metabolite A (for details see main text). Right: concentrations of metabolites A and D .

**FIGURE 16:**
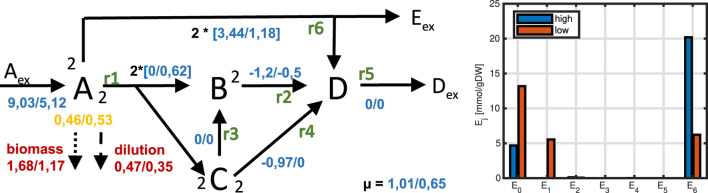
Left: Flux maps for high and low substrate input. For metabolite A all terms that appear in the steady state equation for the concentration of A are given. Values for all fluxes (blue numbers), for drain into biomass, and dilution are shown (red numbers). Yellow numbers indicate the concentration of metabolite A . Small black numbers represent the stoichiometry. Right: Enzyme allocation for high and low substrate.

In the network, the demand is split in several parts. Metabolite A is the center of the network because it is the first metabolite in the network. Hence, it must feed all other metabolites as well the direct drain to biomass. In the left plot in Figure 15, the demand bars show that main part of the incoming fluxes are used for the network demands (yellow $r_{6}$ and red $r_{1}$ part) while the flux directly into the biomass (magenta) is smaller. Since the concentration is small in comparison to the units, the dilution term (green) is very small. To understand, why in the low mode a shift of the allocation of enzymes from $E_{6}$ to $E_{1}$ is observed (Figure 14), a look on the concentrations of A and D reveals also a change here. Since reaction $r_{1}$ is reversible, in the high mode, the situation is not favorable enough because A is lower (and D is higher); in contrast, in the low mode, A is higher (and D is lower)that prevent that D alone feed the other metabolites in the network and, consequently, $r_{1}$ comes into play to feed the network “from the other side” (see Figure 16, left plot). In this way, for example, the demand on metabolite C is satisfied via two complete different ways; in the high mode with $r_{6}$ and the reverse $r_{4}$ reaction and in the low mode directly via $r_{1}$ . The dilemma is also illustrated with the enzyme allocation plot in Figure 16, right. For a low concentration outside, more enzyme must be spend for substrate uptake (compare bars for $E_{0}$ ). However, the complete amount of enzyme is restricted, and for reaction $r_{6}$ , enzyme is not available in high amounts. Consequently, enzyme spend to reaction $r_{6}$ is reduced and enzyme for reaction $r_{1}$ is significantly increased in the low mode. The example also demonstrates the high sensitivity of the system; while the change in the concentration of A is marginal ( ∼ 7%) it goes along with a dramatic change in the proteome pattern.

## 3 Summary

The provided examples demonstrate an increasing complexity starting from a detached network with a couple of reactions and ending with a prototype of a cellular system with metabolism and macromolecular synthesis as well as kinetics for the metabolic network. While the simple network structure without biomass reactions must be scaled by the incoming flux, the last examples provide self-contained models. Thermodynamic considerations play an important role for the outcome of the simulations, but could be considered in the kinetic reaction expressions directly, bridging pure fluxes with metabolite concentrations.

**FIGURE 17:**
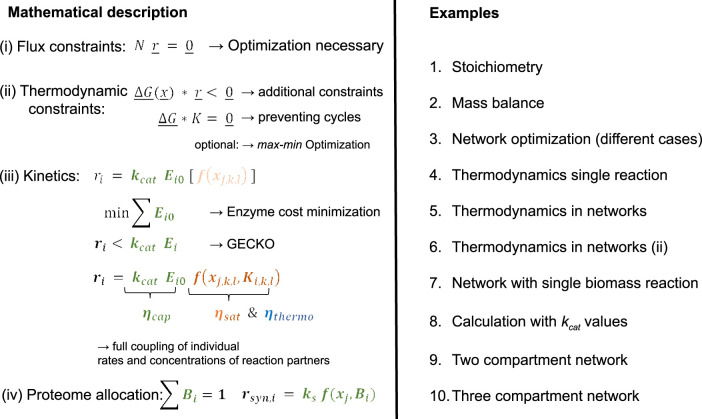
Overview equation systems and examples.

Starting from flux balance analysis, using different objective functions to finally a three macromolecular unit model, increasing levels of complexity are developed and demonstrated by numerical examples. A final summary of basic equations and examples are given in Figure 17.

## Data Availability

The datasets presented in this study can be found in online repositories. The names of the repository/repositories and accession number(s) can be found below: https://sourceforge.net/projects/fba-to-self-contained-models/.

## Footnotes

1 Please note, that symbol n is also used for the molar number of a component.

2 Conversion between both number can be done with the cellular density ρ=330g/l , see (Kremling, 2021).

3 For basic properties of equation systems, we refer to the Supplementary Appendix.

4 In the cited publication, 11 objective functions are analysed for *E. coli* under different environmental conditions. However, in completely different settings or when considering growth of different species in mixed cultures, it might be challenging to find a suitable objective function.

5 The concept of overflow addresses the excretion of metabolites from central metabolism for example during high growth rates. It is observed for *E. coli* that the uptake of carbohydrates and oxygen consumption are not perfectly balanced, that is, for high rates of glucose uptake, the cell cannot use the carbon for growth due to the low availability of enzymes in the respiration chain. The surplus carbohydrates are converted for example into acetate that is excreted.
