# Supplementary material for: From flux analysis to self contained cellular models
Source: Front Syst Biol. 2025 Aug 22;5:1546072. doi: 10.3389/fsysb.2025.1546072 (PMC12411176; doi:10.3389/fsysb.2025.1546072)
Supplement: Supplementary file 1 [file DataSheet1.pdf]

Appendix

From flux analysis to self contained cellular models

Author & Address: Andreas Kremling, Systems Biotechnology, School of Engineering & Design, Technical University of Munich, Boltzmannstr. 15, Garching b. München;
Mail: a.kremling@tum.de

Abstract of the manuscript

Mathematical models for cellular systems have become more and more important for understanding the complex interplay between metabolism, signalling, and gene expression. In this manuscript, starting from the well-known flux balance analysis, tools and methods are summarised and illustrated by various examples that describe the way to models with kinetics for individual reactions steps that are finally self-contained. While flux analysis requires known (measured) input fluxes, self-contained (or self-sustained) models only get information on concentrations of environmental components. Kinetic reaction laws, feedback structures, and protein allocation then determine the temporal output of all intracellular metabolites and macromolecules. Emphasis is placed on (i) mass conservation, a crucial system property frequently overlooked in models incorporating cellular structures like macromolecular structures like proteins, RNA, and DNA, and (ii) thermodynamic constraints which further limit the solution space. Matlab Live Scripts are provided for all simulation studies shown and additional reading material is given in the appendix.

Keywords: mass balances / flux balance analysis / dynamic modeling / coarse-grained modeling / thermodynamics / enzyme cost minimization / max-min driving force / resource allocation / enzyme kinetics

Notation

Here we summarize the main variables used in the tutorium:

m_X	biomass g/l	n	mol number of a component mol
c	concentration mol/gDW	μ	specific growth rate $1/h$
A	name of a component	γ	stoichiometric factor $[-]$
w_A	molecular weight g/mol	r	reaction velocity (specific) $mol/gDWh$
N	stoichiometric matrix	ΔG	Gibbs energy J
	l components and q reactions		

Basic properties of linear equation systems

A linear equation system is given by $A\,\underline{x} \;=\; \underline{b}$ with x_i are all the unknowns that are stored in a vector. The aim is to find the x_i values in such a way that the equation system is fulfilled. For the matrix, the rank is the most important number that characterizes the solution space. The rank r is the number of the linear independent rows / columns (it is equivalent to look for rows or columns). If a linear combination of all vectors of matrix A in the form

$$\lambda_1\ \underline{a}_1\ +\ \lambda_2\ \underline{a}_2\ +\ \cdots\ =\ 0 \tag{1}$$

has the single solution $\lambda_1 = \lambda_2 = \cdots = 0$, then all vectors are linear independent.

The solution depend on the structure of matrix A. The following list summarizes possible cases:

- Matrix A is square $(n \times n)$ and has rank n, then, there exist an inverse matrix A^{-1} and the solution of the equation system is $\underline{x} = A^{-1}\ \underline{b}$.
- Matrix A has l rows and q columns, $l > q$ and has rank q, that is, all columns are linear independent, then the solution of the equation system is $\underline{x} = A^{\#}\ \underline{b}$ with the so called pseudo inverse $A^{\#} = (A^T\ A)^{-1}\ A^T$.
- Matrix A has l rows and q columns, $l < q$ and has rank l, that is, all rows are linear independent, then the solution of the equation system is $\underline{x} = A^{\#}\ \underline{b}\ +\ K\ \underline{c}$ with the pseudo inverse $A^{\#} = A^T\ (A\ A^T)^{-1}$, the null space K of A and an arbitrary vector $\underline{c}$. In this case, the number of solutions is infinite since combinations of free selectable entries in vector $\underline{c}$ generate also solutions. A vector $\underline{k}$ is a member of the null space of matrix A, if $A\,\underline{k}\ =\ 0$ is valid. To find out the dimension of the null space, the following property hold: $dim(Null(A))\ =\ q\ -\ r$ with q is the number of columns of A.

  This is the situation for most applications in flux analysis and also in the networks presented in the main text. To find a meaningful solution (in the sense of physiologically plausible), the equation system is combined with an objective function that finds the best solutions with respect to the maximization or minimization of this function.

Elementary flux mode

Elementary flux modes are flux vectors that fulfill the following two conditions: (i) they are linear combinations of the null space of the stoichiometric matrix and (ii) they are not decomposable, that is, there is no subset of elements of the vector that fulfills property (i). For larger networks, sophisticated algorithms are required for a proper solution. The following example shows all null space vectors and elementary flux modes. The

stoichiometric matrix is as follows (with only the two internal metabolites A and B and four reactions):

$$N = \begin{pmatrix} 1 & -1 & -1 & 0 \\ 0 & 1 & 2 & -1 \end{pmatrix} \qquad (2)$$

For the simple network, the dimension of the null space is given by the difference of the number of columns of N and the rank of N, that is $\dim(null(N)) = 4 - 2 = 2$. Figure 1 shows the two null space vectors and two linear combinations that are elementary flux modes (in our case, the null space vectors itself are also elementary flux modes). Furthermore, two additional linear combinations are given, that are not elementary flux modes. Please note, that the shown vectors result in different values for yield coefficients with respect to C_{ex} and B_{ex}.

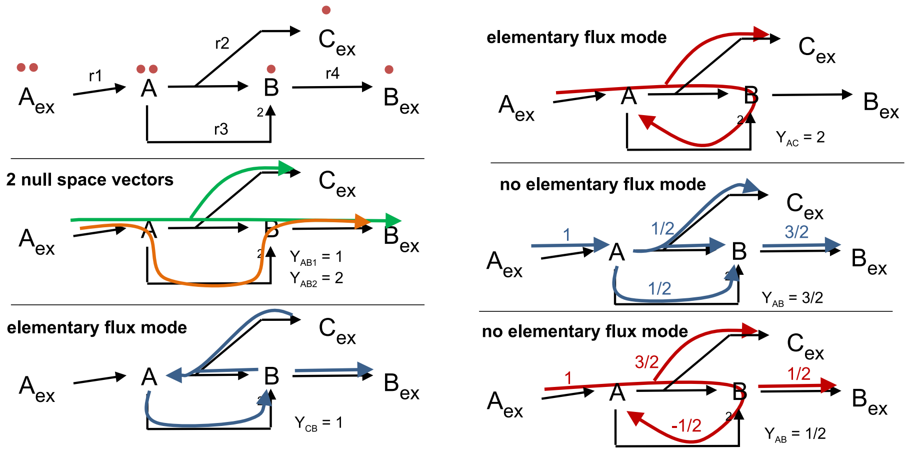

Figure 1: Null space vectors, elementary flux modes and further linear combinations for matrix N.

Elementary flux modes for the models with biomass equations

Two model variants are given that include biomass production; one with the traditional FBA approach with one additional reaction (given as flux that is leaving the system) and one with three units. Also here, for the matrix $M\,N$ (with M includes the dilution term), elementary flux modes can be calculated. For a introduction into elementary flux modes, see [Zanghellini et al., 2013]. The following tables provides elementary flux modes with substrate A_{ex} uptake and biomass production – all other modes are not given here, but can be determined easily with an appropriate algorithm. Please note that all modes are scaled to the uptake of $10\,mmol/gDW\,h \equiv 1$. Growth rate and sum of fluxes for the metabolic network are given also.

Table 1: Columns vectors with elementary flux modes for the model with a single biomass flux (FBA). The entries in the fist line corresponds to an uptake rate of $10\,mmol/gDW\,h$.

$$\begin{array}{cccccc}
1 & 1 & 1 & 1 & 1 & 1 \\
\frac{3}{8} & \frac{3}{8} & 0 & \frac{9}{46} & \frac{6}{49} & 0 \\
\frac{9}{40} & 0 & -\frac{27}{110} & 0 & -\frac{9}{98} & -\frac{3}{22} \\
\frac{9}{20} & 0 & -\frac{12}{55} & \frac{3}{23} & 0 & 0 \\
0 & \frac{9}{40} & 0 & 0 & 0 & -\frac{6}{55} \\
0 & 0 & 0 & 0 & 0 & 0 \\
0 & 0 & \frac{9}{22} & \frac{9}{46} & \frac{27}{98} & \frac{9}{22} \\
\hline
150 & 150 & \frac{1200}{11} & \frac{3000}{23} & \frac{6000}{49} & \frac{1200}{11} \\
\text{growth rate} & & & & & \\
\frac{3}{2} & \frac{3}{2} & \frac{12}{11} & \frac{30}{23} & \frac{60}{49} & \frac{12}{11} \\
\text{sum of rates } (r_i) & & & & & \\
\text{without biomass} & & & & & \\
\frac{41}{20} & \frac{8}{5} & \frac{52}{55} & \frac{35}{23} & \frac{64}{49} & \frac{64}{55}
\end{array} \tag{3}$$

Table 2: Column vectors with elementary flux modes for the model with macromolecular units. The entries in the fist line corresponds to an uptake rate of $10\,mmol/gDW\,h$.

1	1	1	1	1	1	1	1	1
$\frac{151}{400}$	$\frac{151}{400}$	$\frac{151}{400}$	0	$\frac{90}{461}$	$\frac{123}{979}$	$\frac{3}{8}$	0	0
$\frac{183}{800}$	0	$\frac{1}{200}$	$-\frac{135}{551}$	0	$-\frac{171}{1958}$	0	0	$-\frac{147}{1102}$
$\frac{179}{400}$	$-\frac{1}{100}$	0	$-\frac{123}{551}$	$\frac{57}{461}$	0	0	$\frac{147}{551}$	0
0	$\frac{183}{800}$	$\frac{179}{800}$	0	0	0	$\frac{171}{784}$	$-\frac{135}{551}$	$-\frac{123}{1102}$
0	0	0	0	0	0	$-\frac{3}{196}$	0	0
0	0	0	$\frac{453}{1102}$	$\frac{183}{922}$	$\frac{537}{1958}$	0	$\frac{453}{1102}$	$\frac{453}{1102}$
150	150	150	$\frac{60000}{551}$	$\frac{60000}{461}$	$\frac{4312691165127517}{35184372088832}$	$\frac{7500}{49}$	$\frac{60000}{551}$	$\frac{60000}{551}$
150	150	150	$\frac{60000}{551}$	$\frac{60000}{461}$	$\frac{4312691165127517}{35184372088832}$	$\frac{7500}{49}$	$\frac{60000}{551}$	$\frac{60000}{551}$
120	120	120	$\frac{48000}{551}$	$\frac{48000}{461}$	$\frac{96000}{979}$	$\frac{6000}{49}$	$\frac{48000}{551}$	$\frac{48000}{551}$
growth rate								
$\frac{3}{2}$	$\frac{3}{2}$	$\frac{3}{2}$	$\frac{600}{551}$	$\frac{600}{461}$	$\frac{1200}{979}$	$\frac{75}{49}$	$\frac{600}{551}$	$\frac{600}{551}$
sum of rates (r_i)								
$\frac{1643}{800}$	$\frac{1277}{800}$	$\frac{257}{160}$	$\frac{1039}{1102}$	$\frac{1399}{922}$	$\frac{1285}{979}$	$\frac{1237}{784}$	$\frac{1579}{1102}$	$\frac{1285}{1102}$

(4)

Thermodynamic in networks

In a strict sense, flux maps with zero entries in fluxes through a cycle could be thermodynamically inconsistent. This is the case, if two different pathways from a component A to C exist; one direct and a second one via component B as can be seen in Figure 2. If there is no flux in one of the two pathways, the components are in equilibrium, therefore, also for the second pathway, this holds true, and consequently there is also a zero flux. For the small example, showing in the Figure, three cases are shown, and only case 3 is a valid flux map, since in the other cases, fluxes from component A via B to C or directly to C are not possible due to the condition $K^T \underline{\Delta G}$ with K is the null space of the internal reaction network.

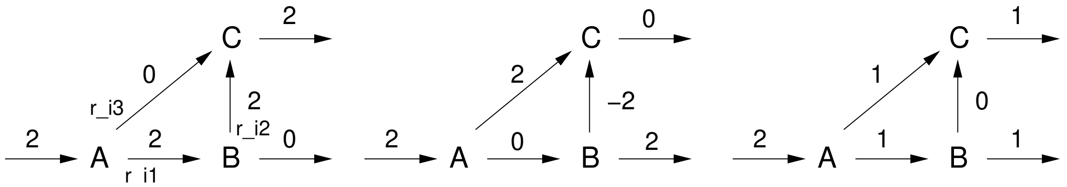

Figure 2: Network with cycle and three flux maps; only the third flux map is thermodynamically feasible in a strict sense.

For the given network, three reactions build a cycle with the null space vector $K^T = [1, 1, -1]$. Therefore, condition $K^T \underline{\Delta G} = 0$ can be formulated for the cases:

(i) $r_{i3} = 0$, it follows $\Delta G_1 + \Delta G_2 = 0$. This conditions requires that the corresponding fluxes must have the opposite sign, one must be positive, and the second one negative. This is not the case in the flux map.

(ii) $r_{i1} = 0$, it follows $\Delta G_2 - \Delta G_3 = 0$. This conditions requires that the corresponding fluxes must have equal signs. This is not the case in the flux map.

(iii) $r_{i2} = 0$, it follows $\Delta G_1 - \Delta G_3 = 0$. This conditions requires that the corresponding fluxes must have equal signs. This is the case in the third flux map.

As described in the main text, these cases cannot be described with the procedure, since the values for the Gibbs energy are not allowed to be zero. Here, we present a procedure that allows us to check, if a consistent flux map can be found, starting from the set of equations presented in the main text (Eq. (36) therein). Let us assume that we obtain a flux map by solving the mixed integer program and one of the internal fluxes in a cycle is zero. Then we consider only those reactions with fluxes $\neq$ zero and we solve the equation system:

$$\begin{pmatrix} -(M + \epsilon)_\sim & -I_\sim \\ (M + \epsilon)_\sim & I_\sim \end{pmatrix} \begin{pmatrix} \underline{z} \\ \underline{\Delta G} \end{pmatrix} \leq \begin{pmatrix} -\epsilon \\ M \end{pmatrix} \tag{5}$$

The notation is as follows: with $\sim$ we consider the same matrices as before, but canceling out columns corresponding to zeros fluxes. This is necessary since in case of zero fluxes, the corresponding integer variables z_i could either be positive or negative. Solving equation (5) re-adjust the integer variables and the values for the Gibbs energy. With the solution we than check, if the sign of the (given) flux values is in agreement with the newly determined ΔG values; that is, if a specific reaction rate was positive, the corresponding ΔG value must be negative and the condition $K^T\ \underline{\Delta G}$ must hold true.

Matlab Live Script and other tools

The matlab live scripts and the pdf from the scripts can be found elsewhere:

`https://sourceforge.net/projects/fba-to-self-contained-models/`

The following tools are integral to systems biology, providing researchers with robust platforms for modeling and simulating cellular processes. Each offers unique features tailored to different aspects of metabolic network analysis and offer methods beyond the method spectrum provided here.

COBRA Toolbox

The COBRA Toolbox is a comprehensive MATLAB-based suite for constraint-based modeling of biochemical networks. It supports methods like Flux Balance Analysis (FBA), Flux Variability Analysis (FVA), and gene knockout simulations. The latest version (v3.0) includes enhancements for multi-scale modeling, integration with omics data, and improved solvers for nonlinear and thermodynamic analyses Heirendt and 53 further authors [2019].

`https://opencobra.github.io/cobratoolbox/stable/index.html`

COBRApy

COBRApy is a Python implementation of the COBRA Toolbox, offering an object-oriented interface for model construction and analysis. It supports reading and writing models in formats like SBML, MATLAB, and JSON, and includes functionalities for FBA, FVA, and gene deletion analysis. COBRApy is widely used for its flexibility and ease of integration into Python-based workflows Ebrahim et al. [2013].

`https://opencobra.github.io/cobrapy/`

COBREXA

COBREXA (COBRA Extension for eXtended Analysis) is a Julia-based software designed for scalable and efficient constraint-based modeling. It is optimized for high-performance computing environments, enabling the analysis of large-scale metabolic networks. COBREXA supports parallel computations and is particularly suited for large datasets and complex simulations

`https://lcsb-biocore.github.io/COBREXA.jl/stable/`

Additional reading material

A list with additional reading material is provided that includes general text books in the field as well as publications that bridge the gap from the simple models used here to genome scale models.

<u>Books</u>

B. O. Palsson. Systems Biology: Properties of Reconstructed Networks. Cambridge University Press 2006.
Standard text book for constraint based models.

B. O. Palsson. Systems Biology: Simulation of Dynamic Network States. Cambridge University Press 2011.
Second book from the same author with focus on dynamical systems.

U. Alon: An Introduction to Systems Biology. Chapman & Hall, new edition from 2019.
The book focus on network properties, and provides tools and methods for network and model analysis.

R. Milo. Biology by the numbers. Garland Sciences 2015.
A rich source of quantitative data for mathematical models.

A. Kremling. Mathematical Modeling and Model Analysis. Chapman & Hall 2013.
Introduction into mathematical modelling of cellular systems.

B. P. Ingalls. Mathematical Modeling in Systems Biology: An Introduction. The MIT Press 2013.
A further introduction into modelling techniques.

<u>Publications</u>

On a genome scale level, models become difficult to parametrize due to the high number of kinetic parameters. However, some attempts for organism with a small genome are undertaken to set up such models. Here, three reviews and two publications specialized for a specific organism are provided.

Reviews:

D. A. Cruz and M. L. Kemp. Hybrid computational modeling methods for systems biology. Prog. Biomed. Eng. 4, 2022.

K. Georgouli et al.: Multi-scale models of whole cells: progress and challenges. Frontiers Cell Development Biology 11, 2023.

K. Kaizu and K. Takahashi. Technologies for whole-cell modeling: Genome-wide reconstruction of a cell in silico. Development, growth & differentiation 9, 2023.

Publications:

Bacterium *M. genitalium:* J. R. Karr et al.: A Whole-Cell Computational Model Predicts Phenotype from Genotype. Cell 150, 2012.

Bacterium *E. coli:* D. N. Macklin et al.: Simultaneous cross-evaluation of heterogeneous *E. coli* datasets via mechanistic simulation. Science 369, 2020.

References

A. Ebrahim, J. A. Lerman, B. O. Palsson, and D. R. Hyduke. COBRApy: Constraints-based reconstruction and analysis for python. BMC Systems Biology, 2013.

L. Heirendt and 53 further authors. Creation and analysis of biochemical constraint-based models using the COBRA toolbox v.3.0. Nature Protocols, 14, 2019.

J. Zanghellini, D. E. Ruckerbauer, M. Hanscho, and C. Jungreuthmayer. Elementary flux modes in a nutshell: Properties, calculation and applications. Biotechnological Journal, 8, 2013.
